# Deletion of the angiopoietin receptor Tie2 enhances proliferation and sprouting of cardiac endothelial cells

**DOI:** 10.1007/s10456-025-10028-2

**Published:** 2026-01-21

**Authors:** Andrey Anisimov, Madeleine H. Lackman, Hellmut G. Augustin, Eero Mervaala, Kari Alitalo, Sinem Karaman

**Affiliations:** 1 Wihuri Research Institute, Helsinki, Finland; 2 Translational Cancer Medicine Program, Faculty of Medicine, University of Helsinki, 00014 Helsinki, Finland; 3 Individualized Drug Therapy Research Program, Faculty of Medicine, University of Helsinki, 00014 Helsinki, Finland; 4 European Center for Angioscience (ECAS), Medical Faculty Mannheim, Heidelberg University, Ludolph-Krehl-Str. 13-17, 68167 Mannheim, Germany; 5 Division of Vascular Oncology and Metastasis, German Cancer Research Center Heidelberg (DKFZ), 69120 Heidelberg, Germany; 6 Department of Pharmacology, Faculty of Medicine, University of Helsinki, 00014 Helsinki, Finland

**Keywords:** Proliferation, Sprouting, Tie2 receptor, Angpt2, Tip cell markers

## Abstract

**Background:**

Endothelial cells (ECs) of the heart proliferate and form new vessels in response to vascular endothelial growth factor (VEGF), but VEGF has not benefited the therapy of cardiac ischemia because of its side effects. Here, we explored if deletion of the vascular steady-state homeostasis maintaining *Tie1* and *Tie2* receptor tyrosine kinases affects the proliferation and sprouting of cardiac ECs.

**Methods:**

We analyzed EC proliferation and histological and immunohistochemical stainings by confocal microscopy, plus scRNA and qPCR analyses of gene expression in the heart, kidneys, and lungs of *Tie1*^*fl/fl*^, *Tie2*^*fl/fl*^, and *Tie1*^*fl/fl*^;*Tie2*^*fl/fl*^ mice, in which vascular endothelial cadherin-driven *CreER*^*T2*^ recombinase was used to delete *Tie1*, *Tie2* or both receptors. These analyses were also performed in mice subjected to transverse aortic constriction (TAC). Boyden chamber assays were performed to assess the migration of cultured ECs in cultures with or without *TIE* receptor silencing.

**Results:**

Genetic deletion of *Tie1*, *Tie2*, or *Tie1/Tie2* in mice increased significantly the proliferation of cardiac but not renal or pulmonary ECs, as measured by EdU incorporation into DNA and quantification of the cell cycle marker cyclin D1. *Tie1/Tie2* or *Tie2* deletion, but not *Tie1* deletion alone, induced EC sprouting in coronary vasculature and expression of endothelial tip cell markers, including expression of the FOXO1-regulated *Angpt2* and *Esm1* genes in cardiac versus kidney or lung ECs. Consistent with these findings, silencing of *TIE2*, but not *TIE1*, in cultured ECs resulted in increased migration of ECs. Similar results were obtained in mice subjected to TAC.

**Conclusion:**

Deletion of *Tie2* alone or together with *Tie1* increases the proliferation and sprouting of cardiac, but not renal or pulmonary ECs, without to neovessel formation in the heart.

**Supplementary Information:**

The online version contains supplementary material available at 10.1007/s10456-025-10028-2.

## Introduction

Cardiovascular diseases are the leading cause of mortality worldwide [1]. Although several treatments are available for primary and secondary prevention of atherosclerosis-associated coronary artery disease, insufficient blood supply to cardiac muscle often necessitates invasive restoration of coronary blood flow [2]. Therefore, alternative methods are continuously sought for treatment of myocardial ischemia. It has been shown that in acute myocardial infarction, clonal expansion of endothelial cells (ECs) occurs in the heart as part of an often insufficient neovascularization process [3]. Cardiac delivery of vascular growth factors via gene therapy has been tried for stimulation of neovascularization in the ischemic heart, but without clinical success [4].

ECs regulate vascular permeability and leukocyte transmigration at intercellular junctions formed by several transmembrane proteins [5]. The TIE1 and TIE2 (also known as TEK) receptor tyrosine kinases form heteromeric complexes that are connected *in trans* across the junctions of neighboring ECs through their angiopoietin (ANGPT) ligands [6–8]. ANGPT1 stabilizes multimeric TIE2/TIE1 complexes, which is an essential mechanism for the maintenance of stable intercellular junctions [6, 7], whereas defective inter-endothelial junctions and loss of pericytes contribute to vessel destabilization [9, 10]. In response to challenges, such as disturbed blood flow or inflammation, TIE2 phosphorylation in ECs is decreased by autocrine expression of ANGPT2, which is a weaker agonist than ANGPT1, and by the endothelial phosphatase VE-PTP [11–13]. Constitutive deletion of *Tie2* impairs vascular stability during embryonic development [14, 15]. Interestingly, cardiac-specific deletion of *Angpt1* was sufficient to phenocopy the *Tie2* deletion phenotype in embryos, suggesting that continuous TIE2 activation is required for vascular stabilization [16, 17]. Furthermore, induction of autocrine ANGPT2 expression in ECs acts as an antagonist of the ANGPT1-TIE2 signaling to induce vessel destabilization during development and in hypoxic or inflamed tissues [12, 18, 19]. Thus, constitutive ANGPT1-dependent activation of TIE2 is important for vascular stabilization. Consistent with this, heterozygous *Tie2*-targeted mice show severe inflammatory stress, vascular leakage, and mortality [20, 21].

Evidence published so far suggests that the loss of *Tie2* expression and thus loss of TIE2 signals, caused by genetic deletion, gene silencing, or during various pathological stresses leads to altered gene expression and phenotypic response, such as attenuation of endothelial barrier function and induced vascular leakage [21]. In the present study, we aimed to further understand organ-specific cardiac functions of *Tie2* and *Tie1* and their possible connection with the poor angiogenic potential of cardiac ECs [22]. We used complete ablation of TIE signaling in endothelium by deleting either *Tie1* or *Tie2* receptor, or both receptors simultaneously in homeostatic or ischemic conditions caused by transverse aortic constriction (TAC), and analyzed EC proliferation, sprouting and vessel growth in the heart, kidneys, and lungs.

## Materials and methods

Detailed experimental procedures are available in the Supplemental Materials and Methods.

## Results

### Deletion of *Tie2* or *Tie1/Tie2* increases endothelial cell proliferation and network complexity in cardiac, but not renal or pulmonary vasculature

**Fig. 1:**
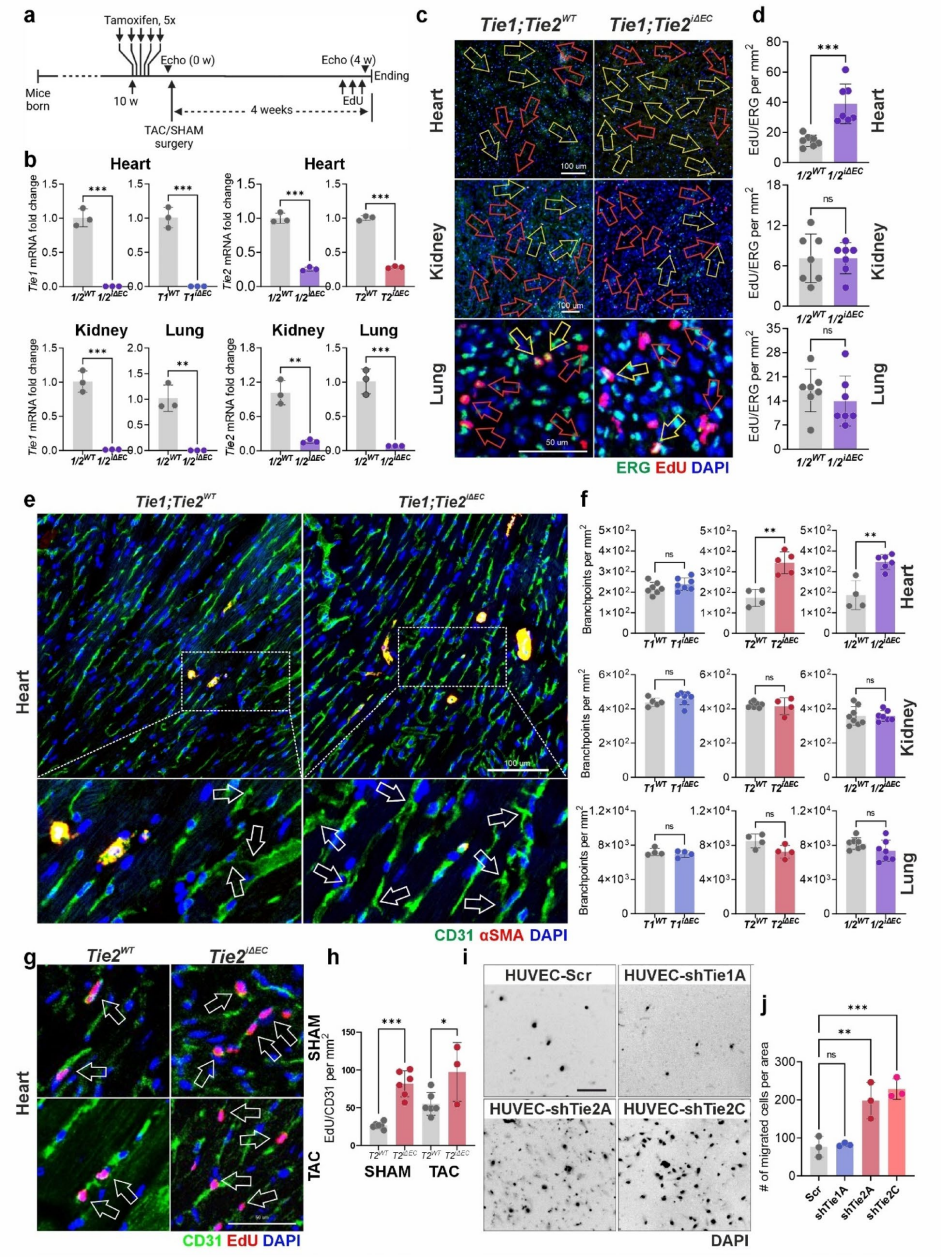
Deletion of *Tie1* and *Tie2* alters proliferation and sprouting of cardiac endothelial cells. **a** Time course of the experiment. **b** qRT-PCR analysis of *Tie1* and/or *Tie2* mRNA levels in total lysates from the target tissues. Marking here onwards: *1/2*^*WT*^ and *1/2*^*iΔEC*^ = *Tie1;Tie2*^*WT*^ and *Tie1;Tie2*^*iΔEC*^, respectively; *T1* ^*iΔEC*^ = *Tie1* ^*iΔEC*^, *T2* ^*iΔEC*^ = *Tie2* ^*iΔEC*^ (*n* = 3 per group). **c** Representative images of immunofluorescently stained sections from the target organs for EdU and ERG. Yellow-outlined arrows indicate EdU^**+**^/ERG^**+**^ nuclei; red-outlined arrows indicate EdU^**+**^/ERG^−^ nuclei. Only images from *Tie1;Tie2*^*iΔEC*^ (vs. control) groups are shown. **d** Quantifications of (**c**). Quantifications of EdU/ERG from the target organs of *Tie1*- or *Tie2*-deleted (vs. control) groups are shown in Fig. S1e (*n* = 7 per group). **e** Representative images of sections from heart immunofluorescently stained for CD31 and αSMA. Arrows indicate examples of the branch points (described in the Materials and methods). Representative images of CD31/αSMA-stained sections of kidney and lung are shown in Fig. S1f and g. **f** Quantification for branchpoints from the images in (**e**) and in Fig. S1f and g from heart, kidney and lung (*n* = 4–8 per group). **g** and **h** Representative images and quantification of cardiac sections from the mice four weeks after SHAM or TAC operations (*n* = 3–6 per group). **i** Experiment using Boyden chamber and HUVECs, silenced for *TIE1* or *TIE2* using lentivirus-delivered shRNA. Representative images of the membranes showing transmigrated DAPI-stained cells on the lower side of the membrane. **j** Quantification of (**i**). Scale bars in (**c**) heart and kidney 100 μm; lung 50 μm; in **e** = 100 μm; in **g** 50 μm (*n* = 3 per group). For statistics in the graphs in (**b**), (**d**) and (**f**) a 2-tailed Student’s *t*-test was used. For statistics on graphs (**h**) and (**j**) one-way ANOVA followed by Dunnett’s *post hoc* test was used. Each dot represents average value per mouse. *P < 0.05; **P < 0.01; ***P < 0.001

To analyze the importance of TIE receptor signals in cardiac blood capillaries, we generated mice carrying *Cdh5-BAC-CreER*^*T2*^ transgene plus conditionally targeted *Tie1*^*fl/fl*^, *Tie2*^*fl/fl*^ alleles or their combination (Fig. S1a). We then deleted the Tie receptors from ECs under homeostatic conditions by administering tamoxifen to 10-week-old male mice. Efficient deletion of both *Tie1* and *Tie2* encoding mRNAs, without deletion of the intervening region in chromosomal DNA, was confirmed by qPCR (Figs. 1a, b and S1b, c and [23]). We also confirmed the lack of compensation of the deleted receptor Tie1 or Tie2 from the partnering receptor Tie2 or Tie1, respectively (Fig. S1d).

To analyze the proliferation effects of *Tie* deletion 4 weeks after tamoxifen administration, mice with an EC-specific deletion of only *Tie1* (*Tie1*^*iΔEC*^), only *Tie2* (*Tie2*^*iΔEC*^), or both *Tie1* and *Tie2* deletions (*Tie1;Tie2*^*iΔEC*^), were injected with six doses of EdU during three consecutive days and terminated 12 h after the last EdU dose. Immunofluorescence stainings and analysis of tissue sections showed that both single and compound deletions led to a significant increase of DNA synthesis in cardiac but not renal or pulmonary ECs, as measured by EdU incorporation into DNA (Figs. 1c, d, S1e). In the hearts of either control or *Tie*-deleted mice, EdU was found to be relatively evenly distributed without accumulation close to the pericardium or the endocardium.

The cardiac vascular network in gene-deleted and control mice, analyzed by quantification of the number of branch points per unit of imaging area, revealed an increase in vascular complexity in the hearts of the *Tie2*^*iΔEC*^ and *Tie1;Tie2*^*iΔEC*^ mice, whereas the difference was not significant in *Tie1*^*iΔEC*^ mice (*p* = 0.2362). There was no significant difference in branch points when comparing gene-deleted kidney or lung in any of the three genotypes analyzed (Figs. 1e, f and S1f, g). The overall vessel-covered area in the cardiac sections was not increased (Fig. S1h). There was no increase in vascular area percentage in renal or pulmonary vessels after deletion of *Tie1*, *Tie2*, or both (Figs. 1f and S1f–h). Furthermore, a small but significant decrease in the vessel-covered area was observed in the lungs of *Tie1;Tie2*^*iΔEC*^ mice (Fig. S1h).

We next analyzed if the effect of *Tie2* deletion in the heart is affected by ischemic conditions induced by TAC, which reduces the diameter of the aorta, leading to pressure overload and adverse cardiac remodeling. *Tie2*^*iΔEC*^ mice and control littermates were treated with tamoxifen and subjected to aortic constriction. Four weeks later, the mice were injected with EdU (twice per day during the last 3 days) and euthanized 12 h thereafter. Analysis of EdU counts associated with CD31-positive ECs revealed an approximately two-fold increase, plus increased branchpoint formation in mice that underwent either SHAM or TAC surgery, but again, there was no significant increase in the overall vessel density (Figs. 1g, h and S2a, b). In echocardiography of the SHAM and TAC groups, *Tie2* deletion did not seem to have any effect. In homeostatic conditions, *Tie1*^*iΔEC*^ did not affect the echocardiography parameters. *Tie2*^*iΔEC*^ affected one echocardiographic parameter, whereas *Tie1;Tie2*^*iΔEC*^ affected five parameters, yet none of the groups, including the TAC group, showed significant differences in ejection fraction (EF) or fractional shortening (FS), indicating no significant changes in myocardial function (Supplemental Table 1). *Tie1* and *Tie2* deletions did not cause hypertrophy or fibrosis in the myocardium (Figs. S2d–f and S3a–c). Furthermore, CD45 + cell accumulation was similar between *Tie1/Tie2*-deleted and control mice, indicating no increase in cardiac inflammation (Fig. S3d–f).

Since vessel branch points develop mainly via vessel sprouting and EC migration in adult mice, we next analyzed if the silencing of *TIE1* or *TIE2* mRNA and the respective proteins affects the migration of cultured ECs. Using a Boyden chamber assay, we tested the migration capacity of human umbilical vein ECs (HUVECs) after silencing *TIE1* or *TIE2* mRNA via lentiviral shRNAs (Fig. S4a). We found that *TIE2*, but not *TIE1* silencing, led to a significant increase in EC migration rate (Fig. 1i, j), which was consistent with the analysis of RNA profiles by hierarchical clustering in HUVECs after *TIE2*, but not *TIE1* silencing (Fig. S4b, c). Furthermore, a striking morphological and kinetic difference was observed between the *shTIE1*, *shTIE2*, and control vector-transfected cells in an EC monolayer wound closure assay. The most conspicuous difference between the two was the elongation of the ECs, accompanied by their improved migration through the 8 μm pores of the Boyden chamber membranes after *TIE2*, but not *TIE1*, silencing (Supplemental videos S1–S3 and Fig. S4d for quantification).

These data indicated that loss of TIE1/TIE2 or TIE2 in mice results in increased proliferation and sprouting of cardiac, but not renal or pulmonary ECs in homeostatic conditions and under ischemic cardiac stress in the TAC model. *TIE2* silencing seemed to increase EC elongation and migration more than *TIE1* silencing in cultured ECs.

### Enhanced expression of endothelial tip cell markers after *Tie1* and/or *Tie2* deletion

**Fig. 2:**
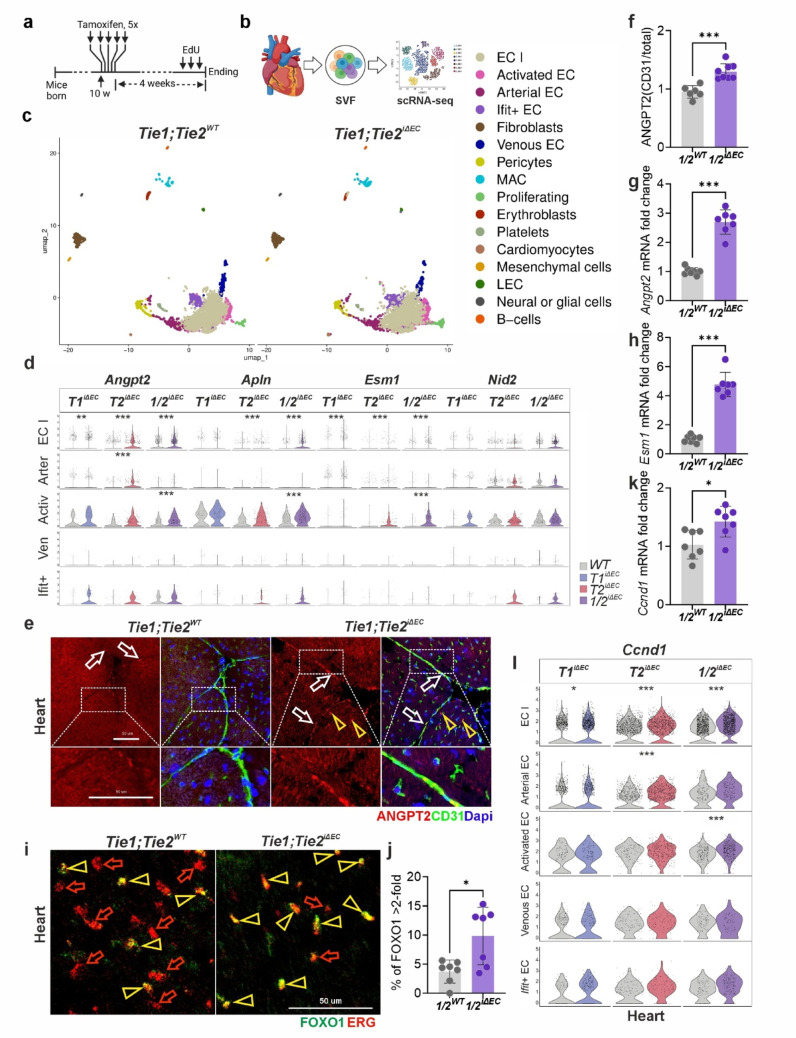
Expression of tip cell markers in cardiac endothelial cells. **a** Time course of the scRNAseq experiment. **b** Schematic of the tissue processing for scRNAseq analysis. **c** UMAP plots (subsampled to 3580 cells each), showing cluster distribution in SVF from the hearts of *Tie1;Tie2*^*WT*^ and *Tie1;Tie2*^*iΔEC*^ mice. Cluster names are shown on the right. The numbers of Mki67^**+**^ERG^**+**^ cells in *Tie1;Tie2*^*WT*^ and in *Tie1;Tie2*^*iΔEC*^ samples are 23 and 72, respectively. Analysis parameters and cluster markers are shown in Supplemental Tables 4 and 5, respectively. More data regarding differentially expressed genes and associated pathway analysis for this tissue and genetic model can be found in Supplemental Tables 10, 15 and 16. For gene signatures of the five major endothelial clusters see Fig. S5a; for UMAP plots of *Tie1*^*WT*^ vs.* Tie1*^*iΔEC*^ and *Tie2*^*WT*^ vs.* Tie2*^*iΔEC*^ hearts see Fig. S5b and c. **d** Violin plots showing relative expression of the four tip cell marker genes *Angpt2*, *Apln*, *Esm1* and *Nid2* in five major EC clusters from the hearts of three genotypes *Tie1;Tie2*^*iΔEC*^ (*1/2*^*iΔEC*^), *Tie2*^*iΔEC*^ (*T2*^*iΔEC*^) and *Tie1*^*iΔEC*^ (*T1*^*iΔEC*^). Pairwise comparisons are made with the corresponding controls (grey). Violin plots are given the same scale of [0, 5]. Asterisks indicate a significant difference between the neighboring violin plots, i.e., the wild-type control (grey) and the *Tie*-deleted one (colored). **e** and **f** Representative images and quantification of ANGPT2 immunofluorescence staining of the heart sections from *Tie1/Tie2*^*WT*^ vs. *Tie1/Tie2*^*iΔEC*^ mice. Quantification represents the ratio of ANGPT2^+^fluorescence intensity (F.I.) within the CD31^+^ areas (calculated as F.I. per µm^2^), divided by the “total” ANGPT2 F.I. from the whole image area (also calculated as F.I. per µm^2^) (*n* = 6–8 per group). **g** and **h** qRT-PCR analysis of *Angpt2* and *Esm1* mRNA expression in the whole heart lysates of *Tie1/Tie2*^*WT*^ vs. *Tie1/Tie2*^*iΔEC*^ mice (*n* = 7 per group). **i** and **j** Representative images and quantification of cardiac sections immunofluorescently stained for FOXO1 and ERG in *Tie1/Tie2*^*WT*^ vs. *Tie1/Tie2*^*iΔEC*^ mice. See Supplemental Materials and Methods section for the details of the quantification (*n* = 7 per group). **k** qRT-PCR analysis of *Ccnd1* mRNA expression in the total heart lysates of *Tie1/Tie2*^*WT*^ vs. *Tie1/Tie2*^*iΔEC*^ mice (*n* = 7 per group). **l** Violin plots showing relative expression of the *Ccnd1* mRNA in five major EC clusters from the hearts of three genotypes *Tie1/Tie2*^*iΔEC*^, *Tie2*^*iΔEC*^ and *Tie1*^*iΔEC*^. Scale bars are 50 μm. Pairwise comparisons are made with the respective undeleted controls, shown on the graph as violins colored in grey. For scRNAseq data, Wilcoxon Rank Sum test was used. For statistics in the graphs in (**f**)–(**h**), (**j**) and (**k**) 2-tailed Student’s *t*-test was used; a Welch’s correction was used for (**j**). Each dot represents average value per mouse. *P < 0.05; **P < 0.01; ***P < 0.001

To analyze how the *Tie* deletion affected the EC transcriptome, we next performed single-cell RNA sequencing (scRNAseq) of EC-enriched fractions from the *Tie1*^*iΔEC*^ vs. control *Tie1*^*WT*^ hearts and stromal-vascular fractions (SVF) of the *Tie2*^*iΔEC*^ and *Tie1;Tie2*^*iΔEC*^ versus their corresponding controls (*Tie2*^*WT*^ and *Tie1;Tie2*^*WT*^, respectively). Figure 2a and b shows the scRNAseq workflow of the cardiac SVF samples after four weeks of gene deletion. We identified 19 distinct cell clusters in cardiac SVF from the *Tie1;Tie2*^*iΔEC*^ mice (Supplemental Table 2). Based on low expression of most genes, damaged cells were excluded, leaving 16 clusters for further analyses (Fig. 2c). Of these, six were identified as endothelial clusters based on commonly known EC markers, such as *Flt1*, *Kdr*, *Cldn5*, and *Pecam1* for blood vascular ECs (BECs), and *Prox1*, *Ccl21a*, and *Flt4* for lymphatic ECs (LECs). The BEC subtypes were further sub-classified as capillary-like EC I (*Aplnr* and *Car4*), arterial (*Hey1* and *Cxcl12*), activated (*Kit* and *Apln*), venous (*Vwf* and *Vcam1*), and Ifit + ECs (*Ifit1* and *Ifit3*) (Fig. S5a, Supplemental Table 2). In line with the immunohistochemical data, scRNAseq analyses confirmed that both single and compound deletions of the Tie receptors increased the number of proliferating cardiac ECs (Figs. 2c and S5b, c [figure legend for the cell numbers]; Supplemental Table 2). We also confirmed that the majority of the proliferating cells in the heart in our genetic models were ECs, as determined by co-expression of *Mki67* and *Erg* (*E*TS-*R*elated *G*ene) markers in our scRNAseq data (Supplemental Table 3). All parameters used for quality control, filtering, normalization, integration and clustering are shown in Supplemental Table 4. Cluster markers for all analyzed genotypes and tissues are listed in Supplemental Tables 5–9.

As sprouting correlates with the expression of tip cell markers, we next investigated if the deletion of *Tie1* and/or *Tie2* genes stimulated the expression of well-known tip cell marker genes, such as *Angpt2*, *Apln*, *Esm1*, and *Nid2* [24, 25]. Indeed, we found a significant upregulation of the tip cell markers, especially in cardiac ECs of the capillary-like cluster, in the *Tie1;Tie2*^*iΔEC*^ and *Tie2*^*iΔEC*^ mice, and to a lesser extent in the *Tie1*^*iΔEC*^ mice (Fig. 2d). Other differentially expressed genes for all analyzed genotypes and tissues are listed in Supplemental Tables 10–14. Immunofluorescence staining was used to confirm increased expression of ANGPT2 in the cardiac sections from the *Tie1;Tie2*^*iΔEC*^ mice (Fig. 2e, f). Furthermore, qRT-PCR analysis of total heart lysates confirmed increased expression of *Angpt2* and *Esm1* RNAs in the *Tie1*^*iΔEC*^, *Tie2*^*iΔEC*^, and *Tie1;Tie2*^*iΔEC*^ mice vs. undeleted control mice (Fig. 2g, h and S5d, e), as well as in the SHAM and TAC-treated mice (Fig. S2c).

The scRNAseq and qRT-PCR analyses of tip cell markers in renal ECs showed *Esm1* upregulation in response to *Tie2* deletion, but only a weak expression of *Angpt2* and *Apln* (Fig. S6a–e). *Tie1* deletion did not lead to upregulation of *Angpt2* or *Esm1* RNA (Fig. S6d, e). Analysis of pulmonary ECs revealed a small, but significant upregulation of *Esm1* RNA in the *Tie1;Tie2*^*iΔEC*^ mice. scRNAseq analysis furthermore revealed no significant alterations of the four tip cell marker genes (Fig. S7a–e). Thus, our results of the tip cell marker gene expression are consistent with the findings of increased branch points in cardiac, but not pulmonary or renal vessels.

### Increased nuclear localization of FOXO1 and ERG in *Tie*-deleted cardiac but not renal or pulmonary ECs

The FOXO1 and ERG transcription factors are abundantly expressed in ECs [26, 27]. Upon activation, they tend to accumulate in the nucleus where they transactivate their target genes. Ligand stimulation of TIE1/TIE2 activates the AKT kinase, which phosphorylates FOXO1, leading to its nuclear exclusion [11, 12]. We found a significant increase of FOXO1 signal in the nuclei of cardiac ECs of the *Tie1*;*Tie2*^*iΔEC*^ mice (Fig. 2i, j), which is consistent with the upregulation of tip cell marker genes (Fig. 2d–h), including *Angpt2*, *Esm1*, which are known FOXO1 target genes [24]. In contrast, no increase of nuclear FOXO1 was observed in Tie1/Tie2-deleted pulmonary ECs (Fig. S8a, b). ERG was markedly accumulated in the nuclei of *Tie1/Tie2*- and to a lesser extent in *Tie2*-deleted cardiac ECs. No increase of nuclear ERG was observed in ECs of *Tie*-deleted kidneys or lungs (Fig. S8c–f).

Transcripts encoding the main G_0_–G_1_ switcher cyclin D1 (*Ccnd1*) were strongly upregulated in major cardiac EC clusters (EC I, Arterial EC and Activated EC; scRNAseq data) of *Tie1;Tie2*^*iΔEC*^ and *Tie2*^*iΔEC*^ mice and in total *Tie1;Tie2*^*iΔEC*^ cardiac mRNA by qRT-PCR (Figs. 2k, l and S9a). Consistent with the EdU staining results, the expression of *Ccnd1* was not significantly altered in any EC subclusters of kidney or lung RNA in the scRNAseq data (Figs. S6f and S7f). As *Ccnd1* is also a direct FOXO1 target [28], its upregulation suggests that the increase in cell proliferation could partly be mediated by FOXO1. We also found that cyclin-dependent kinase 8 (*Cdk8*), involved in cell cycle progression, was strongly upregulated upon *Tie1*/*Tie2* and *Tie2* deletion in cardiac, but not renal or pulmonary ECs (Figs. S6f, S7f and S9b). The results suggest that, unlike the ECs in the kidney and lung, cardiac ECs remain sensitive to TIE signals, in part, via regulation of the intracellular localization of ERG and FOXO1 in ECs.

## Discussion

Our recent studies on the role of the TIE1 and TIE2 receptor signaling in atherosclerosis [23] led us to ask how deletion of the Tie receptor signaling system in mice under homeostatic conditions affects coronary vessels. We show here that, unlike aortic ECs, loss of TIE2 or both TIE1 and TIE2, but not TIE1 alone, in cardiac ECs promotes endothelial proliferation and sprouting. This was an organotypic response that was not observed in the kidneys or the lungs. Surprisingly, also *Tie2* deletion in TAC yielded results corresponding to *Tie2* deletion in homeostatic conditions.

We demonstrated a strong upregulation of *Angpt2* mRNA and ANGPT2 protein expression in the heart upon Tie deletions. As this response was lacking in the kidneys or lungs, it seems to be an organotypic response in cardiac ECs that may depend on specific activation of transcription factors, such as FOXO1 and ETS-1, which both bind to the promoter of the *Angpt2* gene to activate *Angpt2* expression [24, 29]. ANGPT2 in *Tie2*-deleted ECs can bind to and activate α5β1-integrin leading to increased phosphorylation of mitogen-activated protein kinase (MAPK) ERK1/2 [30], which is a master regulator of the G1- to S-phase transition [31]. We show that the GO terms related to ERK1, ERK2 and the MAPK cascade were significantly upregulated upon *Tie1/Tie2*- or *Tie2*-deletion or silencing in cardiac ECs or cultured ECs, respectively (Figs. S4b, c and S9c, d). Other GO terms for all analyzed genotypes and tissues are listed in Supplemental Tables 15–24. Likewise, the lack of *Angpt2* upregulation in renal and pulmonary ECs in response to *Tie* deletion (as we show here) correlated with the lack of ECs' proliferation response in these two organs.

The key endothelial transcription factor ERG was also upregulated in the *Tie1/Tie2* and *Tie2*-deleted cardiac, but not in renal or pulmonary ECs. ERG plays a crucial role in promoting angiogenesis and vascular stability during development and also postnatally. In mature vasculature, ERG also functions to maintain endothelial homeostasis [27]. The increased ERG expression could contribute also to the vascular branching that we observed, and the upregulation of the FOXO1 regulated *Ccnd1* in the *Tie*-deleted cardiac ECs could contribute to increased EC proliferation [28].

It should be noted that our results are consistent with the view that TIE1, which is a weaker tyrosine kinase than TIE2, mainly stabilizes and enhances TIE2 signaling rather than representing a standalone signaling receptor [7, 32]. TIE1 and TIE2 receptors in their transmembrane complex are active in signaling via the PI3-kinase-AKT pathway in steady-state homeostatic ECs of blood vessels [32, 33]. The PI3K-AKT pathway leads to phosphorylation of downstream molecular targets, including the FOXO1 transcription factor, which is thereby sequestered in the cytoplasm where it can be degraded via polyubiquitylation and proteasomal degradation [34]. Unphosphorylated FOXO1 is retained in the nucleus and transactivates target genes such as *Angpt2*, *Esm1* and *Ccnd1* via binding to the promoter/enhancer regions of these genes [24], contributing to the angiogenic events, as shown in this manuscript.

Interestingly, the expression of the tip cell markers *Angpt2*, *Esm1* and *Ccnd1* upon *Tie1*, *Tie2* or *Tie1/Tie2* deletion was low or undetectable in the kidney and lung, respectively. The attenuated response in renal and pulmonary ECs could result from cell- and tissue-type specificity of FOXO1 binding and activation. Specifically, FOXO1 activates a distinct sets of genes in different cell types, as has been shown for ECs and B-cells using chromatin immunoprecipitation (ChIP) analysis. The transcriptional specificity was mediated via the co-binding of ETS and GATA transcription factors in ECs, and ETS and IRF in B-cells [24]. Thus, the intracellular genomic microenvironment in cardiac versus renal and pulmonary ECs could be responsible for the tissue specificity of the FOXO1-mediated transcriptional responses, which could be further investigated in future studies.

It is noteworthy that while we observed increased EdU incorporation following *Tie* deletion in the heart, this did not translate into an overall increase of the cardiac vasculature. Thus, our genetic model did not display a complete angiogenic response. Some angiogenesis-related responses were present, such as enhanced branching, yet we did not detect capillary elongation or fusion. This may not be surprising given that even the master regulator of angiogenesis, VEGF, exhibits a limited efficacy in the heart. For example, in the study by Kocijan et al., VEGF overexpression in skeletal muscle led to the formation of a large number of new capillaries and arterioles. In contrast, the response to the same dose of VEGF in the heart was blunted, yielding only a modest increase in new arterioles [22], suggesting that intrinsic mechanisms within the heart may regulate the angiogenic response.

In conclusion, we discovered that deletion of Tie2 induces cardiac endothelial proliferation and sprouting without vessel formation in the heart. This suggests that additional stimuli maybe ultimately required for angiogenesis. A schematic of our interpretation of the data is shown in Fig. S9e. Further studies should elucidate the therapeutic potential of manipulating the TIE1/TIE2 signaling in the context of therapeutic vascularization.

## Supplementary Information

Below is the link to the electronic supplementary material.

Supplementary Material 1. Supplemental figures S1-S9 and legends, Supplemental table legends, and supplemental materials and methods.

Supplementary Video S1. HUVECs treated with lentivirus-shScramble

Supplementary Video S2. HUVECs treated with lentivirus-shTie1 (clone A)

Supplementary Video S3. HUVECs treated with lentivirus-shTie2 (clone A)

Supplemental Table 1. Echocardiography data of cardiac parameters after Tie1, Tie2 or Tie1/Tie2 gene deletions in homeostatic or ischemic (TAC vs. SHAM) conditions.

Supplemental Table 2. Cell type frequencies in all datasets.

Supplemental Table 3. EC percentages in the clusters of proliferating cells.

Supplemental Table 4. Parameters used for quality control, filtering, normalization, integration, and clustering of the scRNAseq data.

Supplemental Table 5. Cluster markers calculated for the murine Tie1/Tie2 EC double-deletion model heart stromal vascular fraction single-cell RNA sequencing dataset.

Supplemental Table 6. Cluster markers calculated for the murine Tie1 EC deletion model heart EC single-cell RNA sequencing dataset.

Supplemental Table 7. Cluster markers calculated for the murine Tie2 EC deletion model heart stromal vascular fraction single-cell RNA sequencing dataset.

Supplemental Table 8. Cluster markers calculated for the murine Tie2 EC deletion model kidney EC single-cell RNA sequencing dataset.

Supplemental Table 9. Cluster markers calculated for the murine Tie2 EC deletion model lung EC single-cell RNA sequencing dataset.

Supplemental Table 10. Differentially expressed genes in heart stromal-vascular fraction cell types in mice with Tie1/Tie2 EC double-deletion (DKO) versus wild-type mice.

Supplemental Table 11. Differentially expressed genes in heart endothelial cell types and pericytes in mice with Tie1 EC deletion versus wild-type mice.

Supplemental Table 12. Differentially expressed genes in heart stromal-vascular fraction cell types in mice with Tie2 EC deletion (KO) versus wild-type mice.

Supplemental Table 13. Differentially expressed genes in kidney ECs in mice with Tie2 EC deletion versus wild-type mice.

Supplemental Table 14. Differentially expressed genes in lung ECs in mice with Tie2 EC deletion versus wild-type mice.

Supplemental Table 15. Enrichment of downregulated GO terms in heart stromal-vascular fraction cell types in mice with Tie1/Tie2 EC double-deletion versus wild-type mice.

Supplemental Table 16. Enrichment of upregulated GO terms in heart stromal-vascular fraction cell types in mice with Tie1/Tie2 EC double-deletion versus wild-type mice.

Supplemental Table 17. Enrichment of downregulated GO terms in heart endothelial cell types and pericytes in mice with Tie1 EC deletion versus wild-type mice.

Supplemental Table 18. Enrichment of upregulated GO terms in heart endothelial cell types and pericytes in mice with Tie1 EC deletion versus wild-type mice.

Supplemental Table 19. Enrichment of downregulated GO terms in heart stromal-vascular fraction cell types in mice with Tie2 EC deletion versus wild-type mice.

Supplemental Table 20. Enrichment of upregulated GO terms in heart stromal-vascular fraction cell types in mice with Tie2 EC deletion versus wild-type mice.

Supplemental Table 21. Enrichment of downregulated GO terms in kidney ECs in mice with Tie2 EC deletion versus wild-type mice.

Supplemental Table 22. Enrichment of upregulated GO terms in kidney ECs in mice with Tie2 EC deletion versus wild-type mice.

Supplemental Table 23. Enrichment of downregulated GO terms in lung ECs in mice with Tie2 EC deletion versus wild-type mice.

Supplemental Table 24. Enrichment of upregulated GO terms in lung ECs in mice with Tie2 EC deletion versus wild-type mice.

Supplemental Table 25. Antibodies used in the study.

Supplemental Table 26. Primers and Taqman probes.

Supplemental Table 27. TRC clones used in silencing.

## Data Availability

Raw and filtered scRNAseq sequencing files are available on GEO (Accession Number GSE297297; https://www.ncbi.nlm.nih.gov/geo/query/acc.cgi?acc=GSE297297). Other data are provided within the manuscript or supplementary material files.
