# Supplementary material for: Deletion of the angiopoietin receptor Tie2 enhances proliferation and sprouting of cardiac endothelial cells
Source: Angiogenesis. 2026 Jan 21;29(2):13. doi: 10.1007/s10456-025-10028-2 (PMC12823625; doi:10.1007/s10456-025-10028-2)
Supplement: Supplementary file 1 — Supplementary Material 1. Supplemental figures S1-S9 and legends, Supplemental table legends, and supplemental materials and methods. [file 10456_2025_10028_MOESM1_ESM.docx]

**
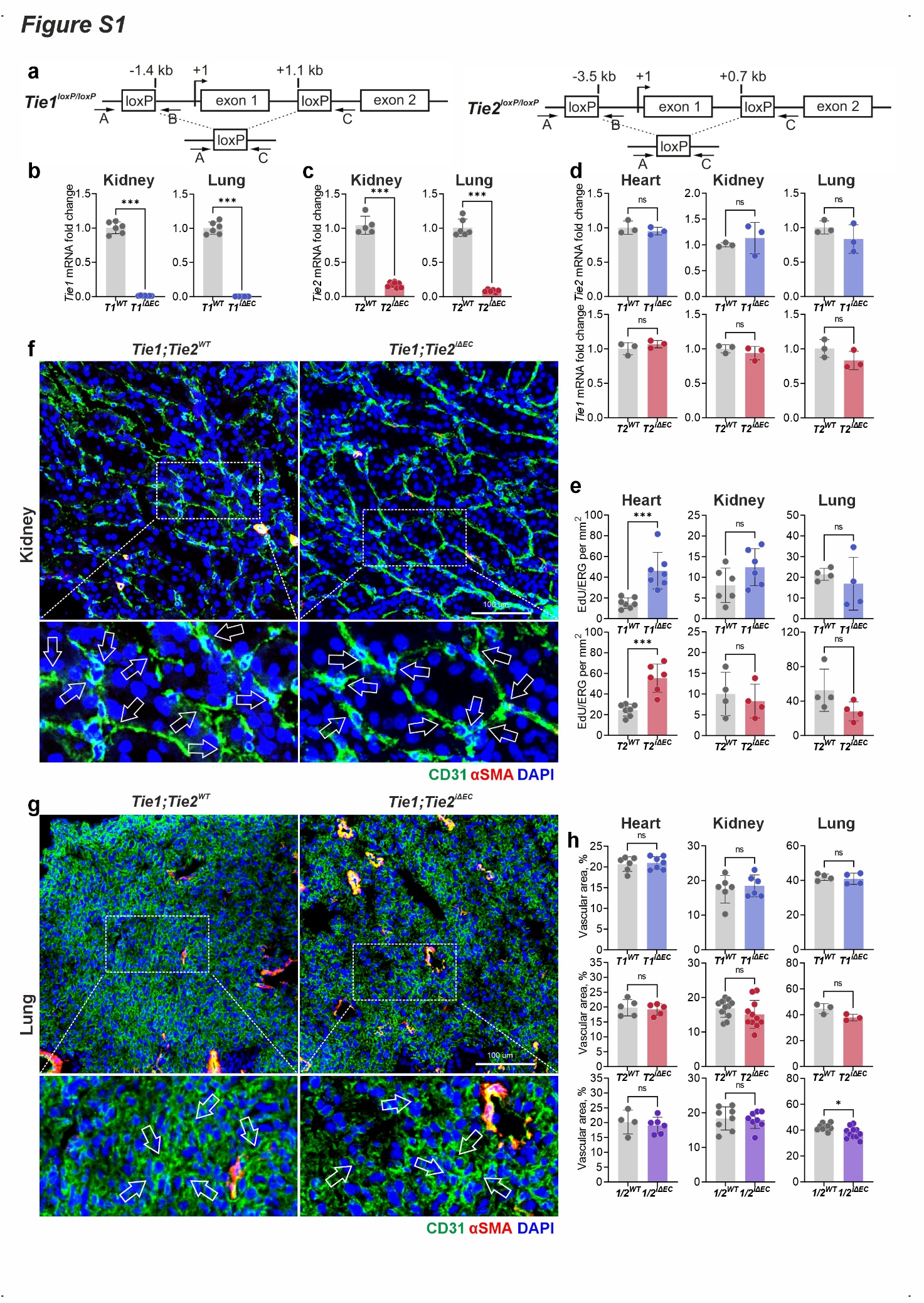
**

**
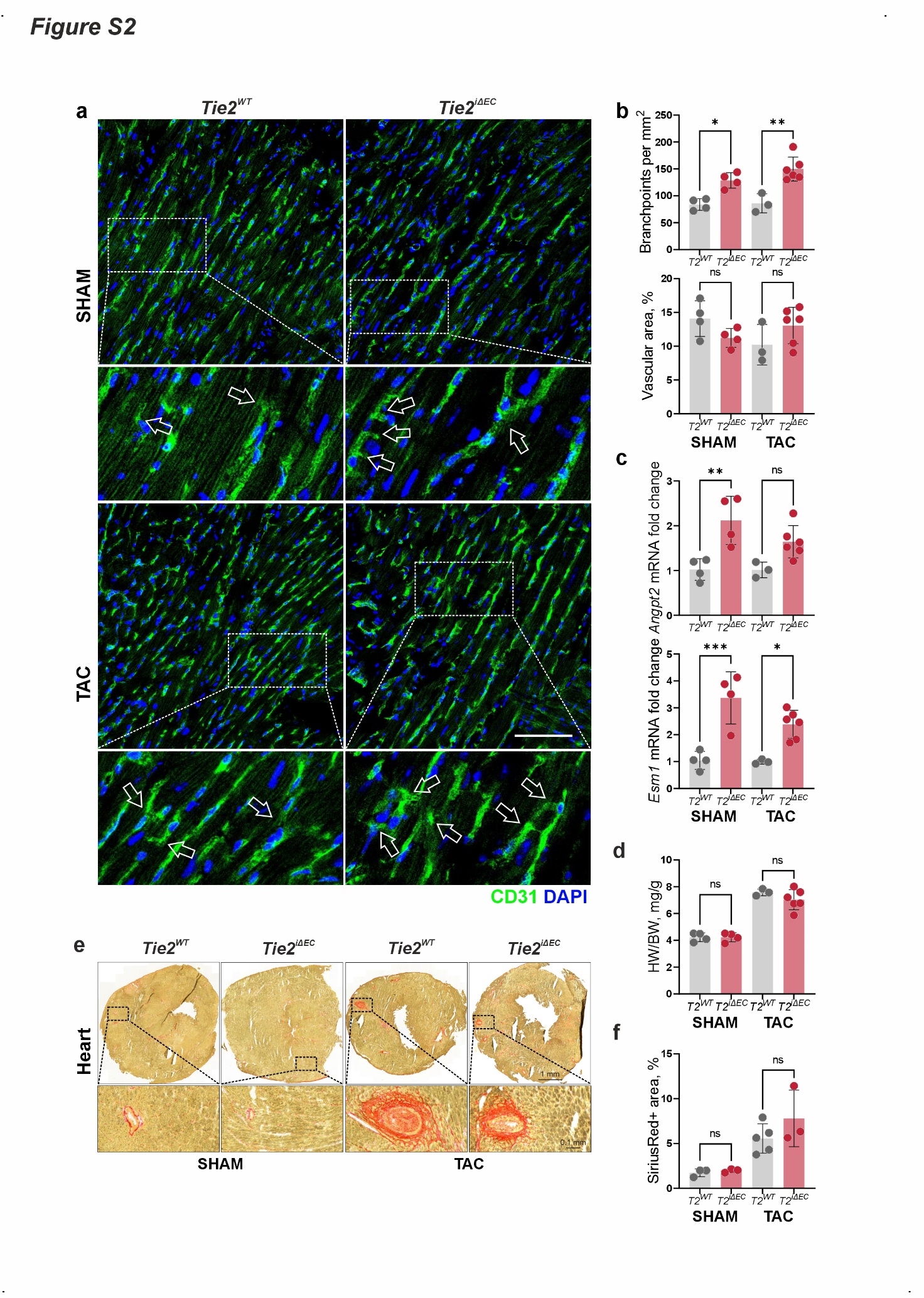
**

**
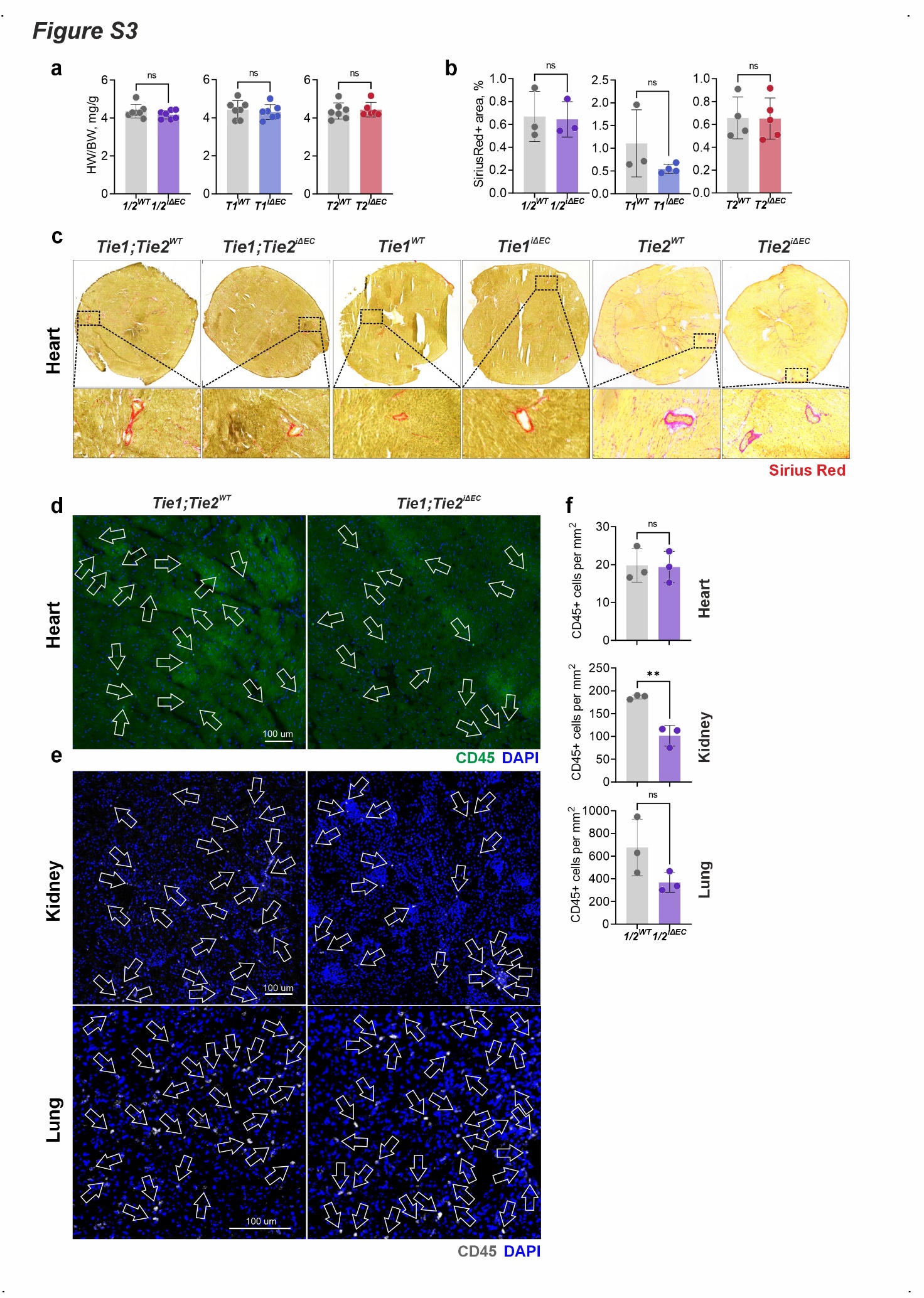
**

**
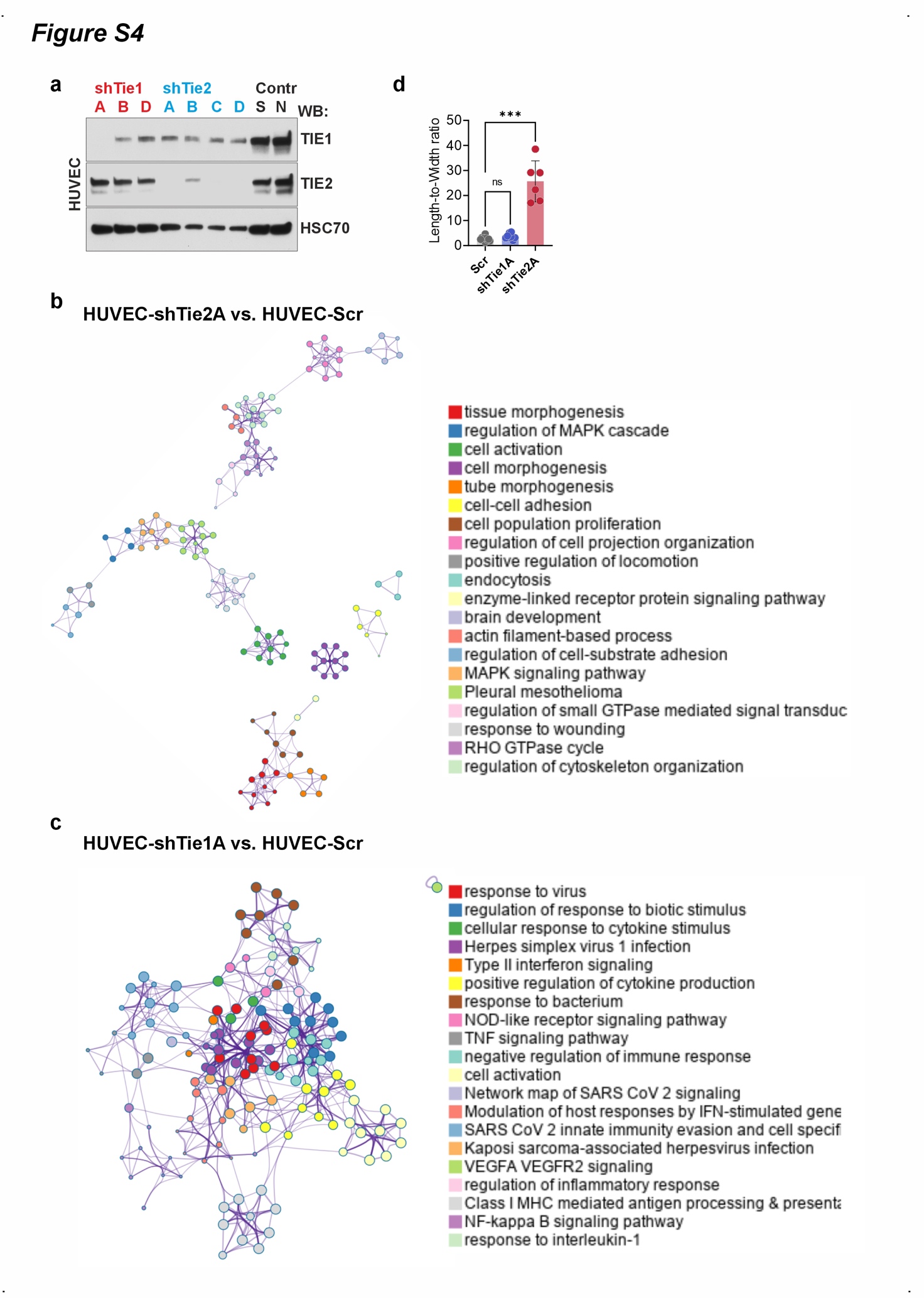
**

**
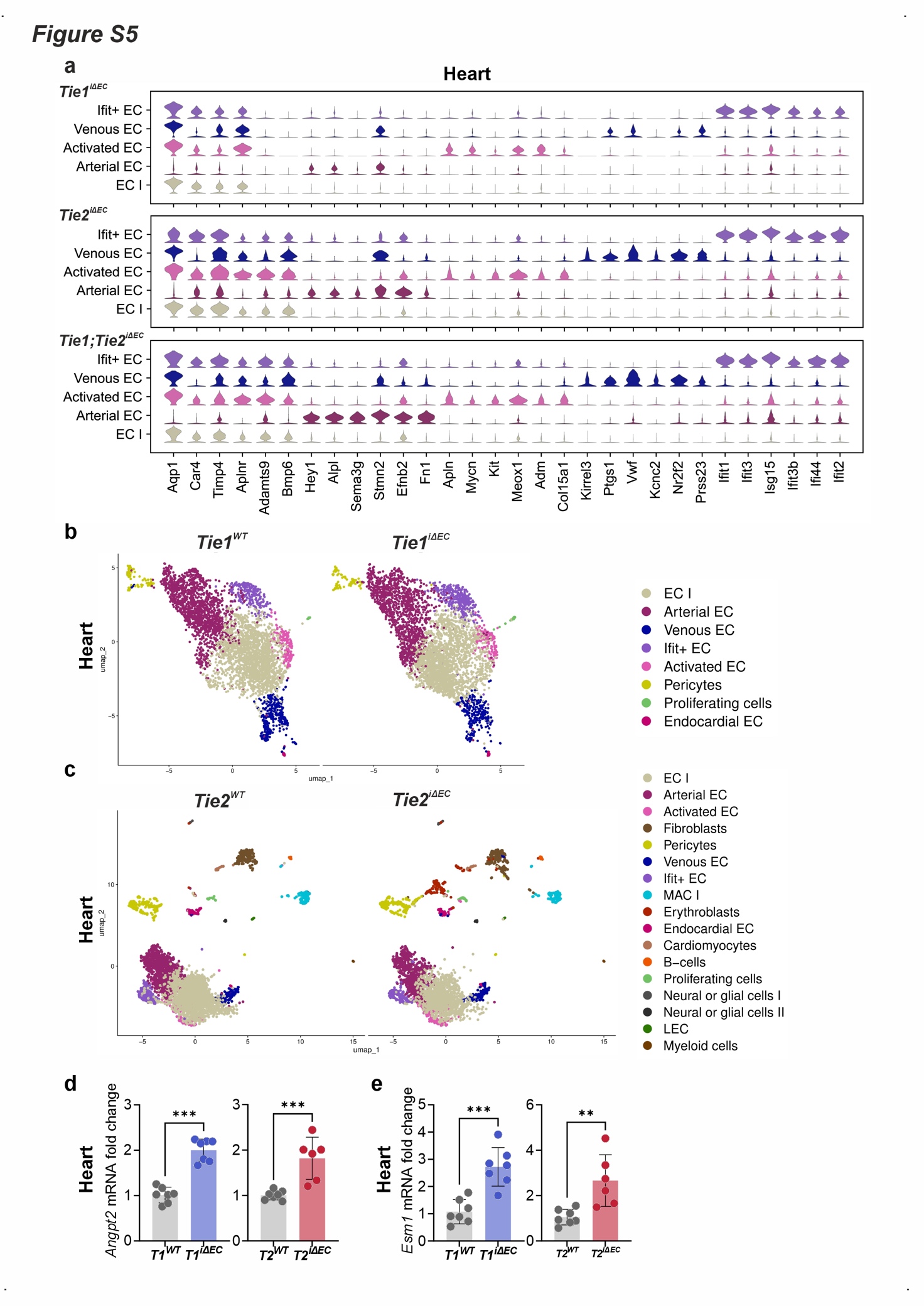
**

**
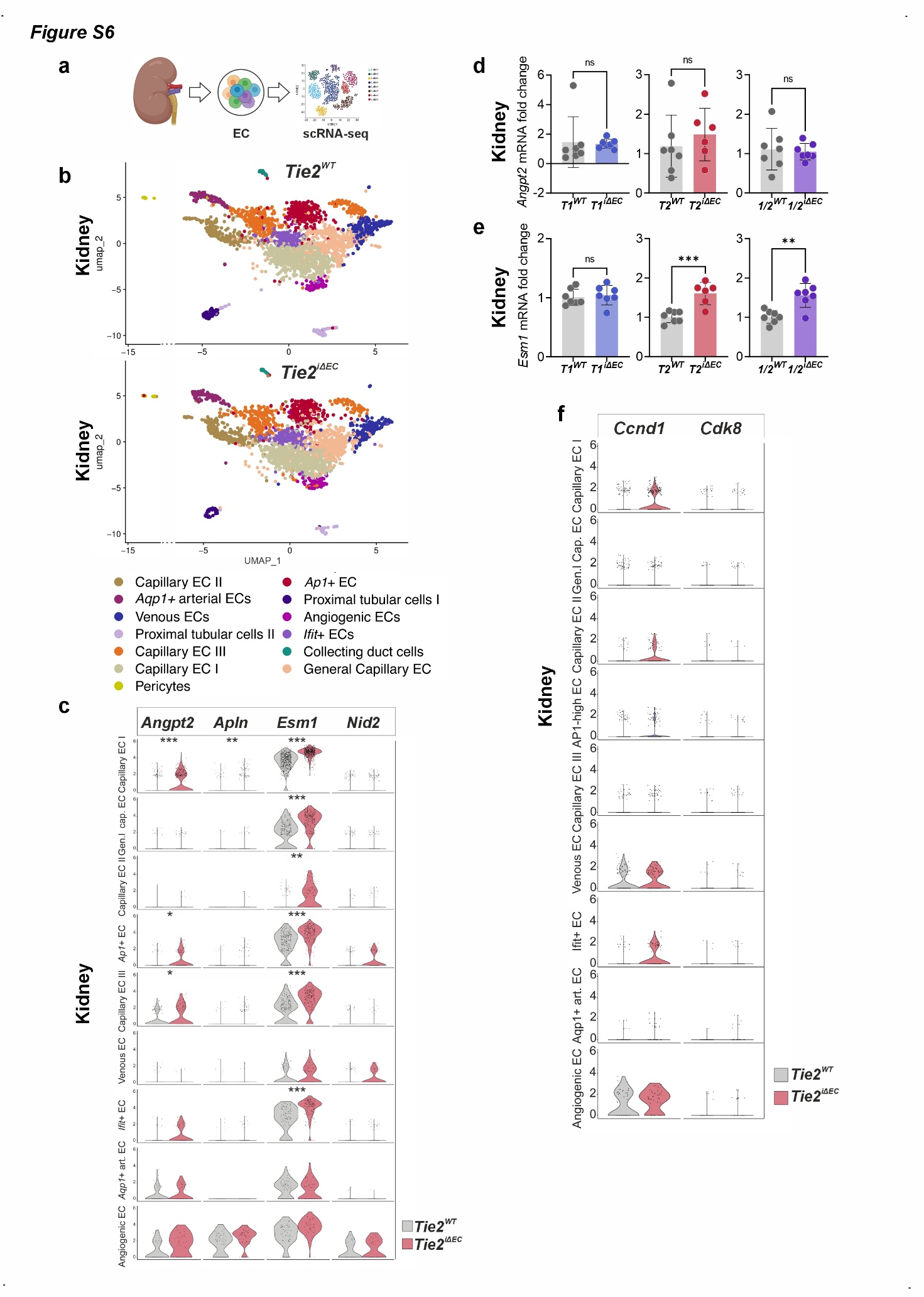
**

**
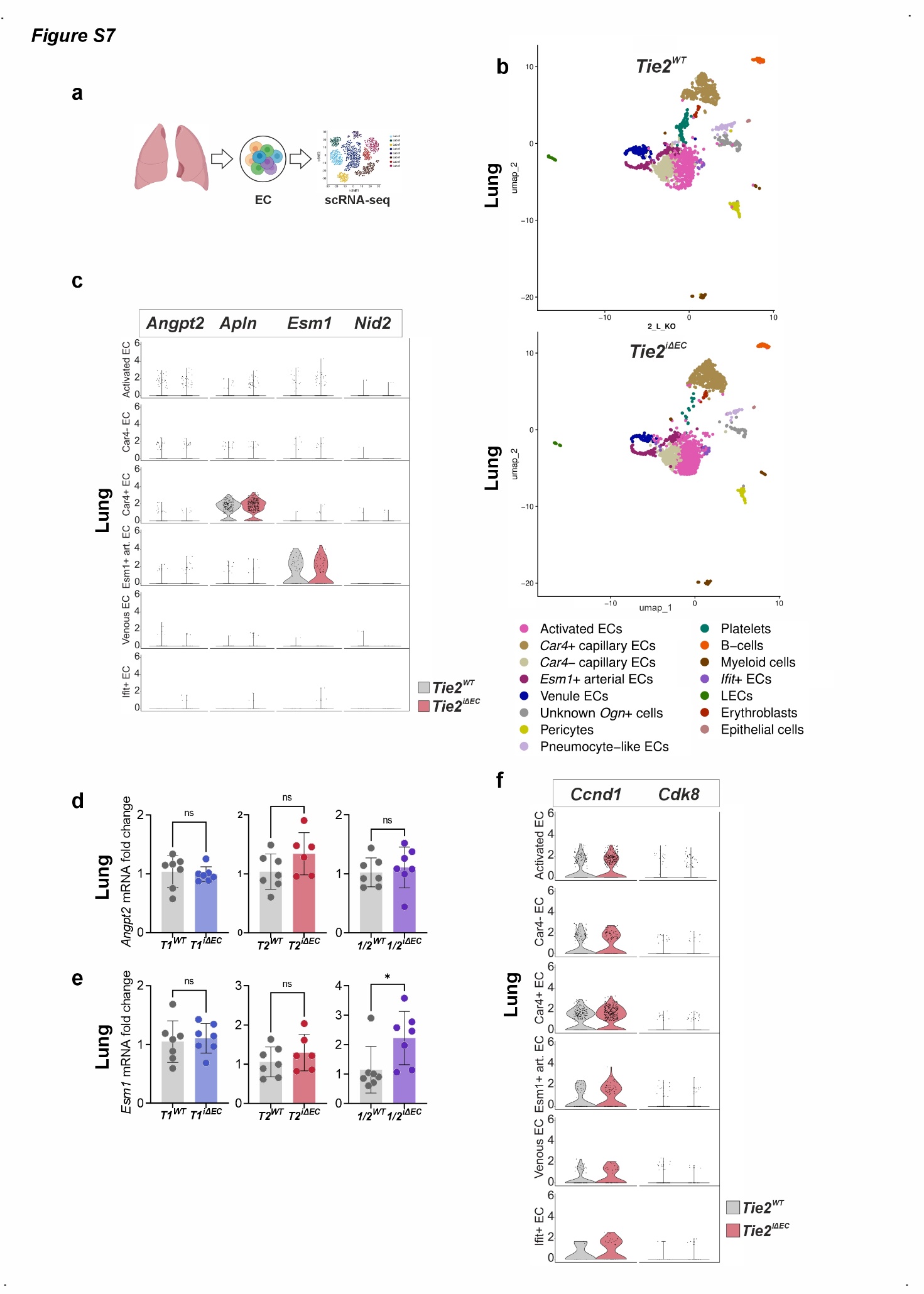
**


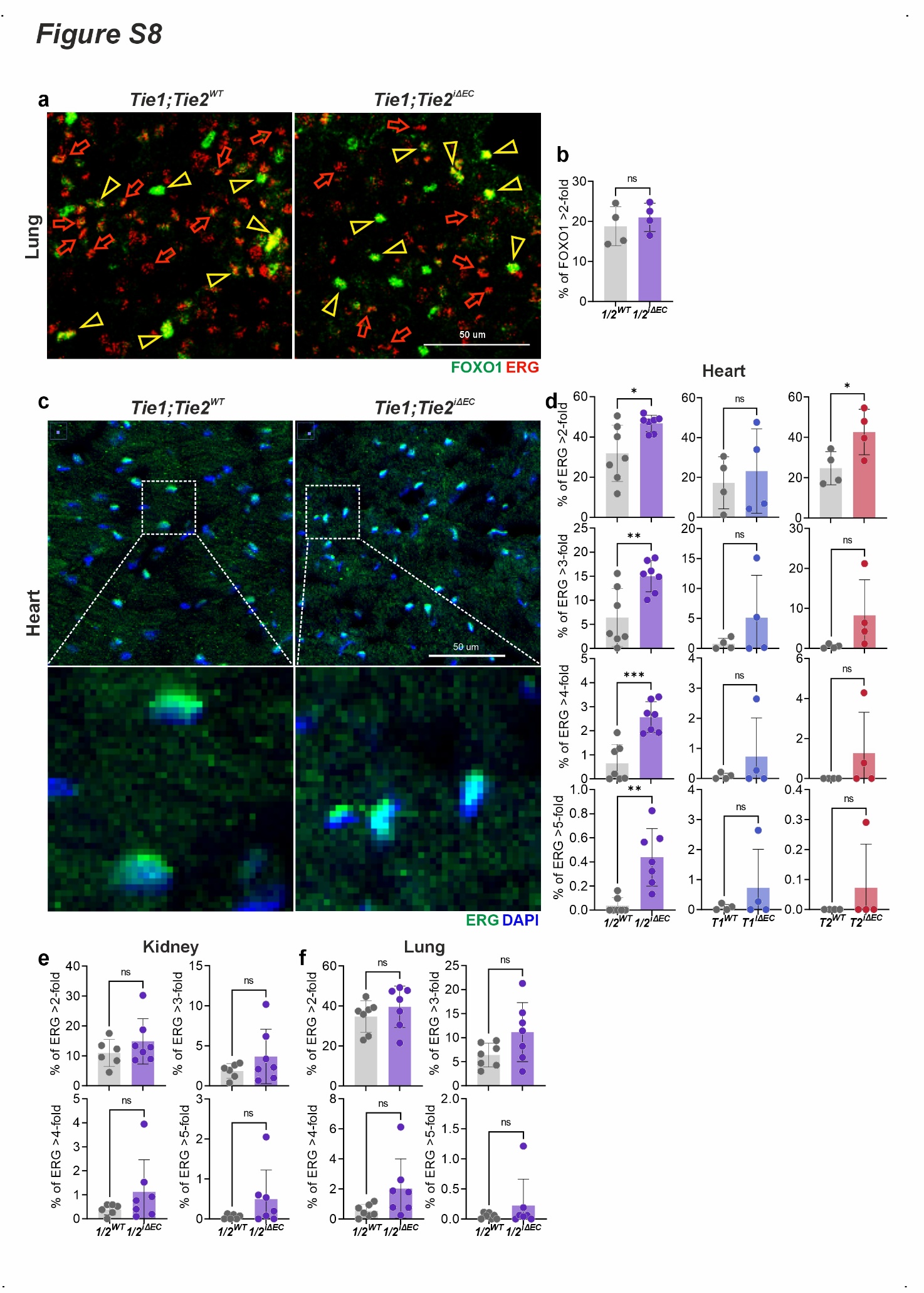


**
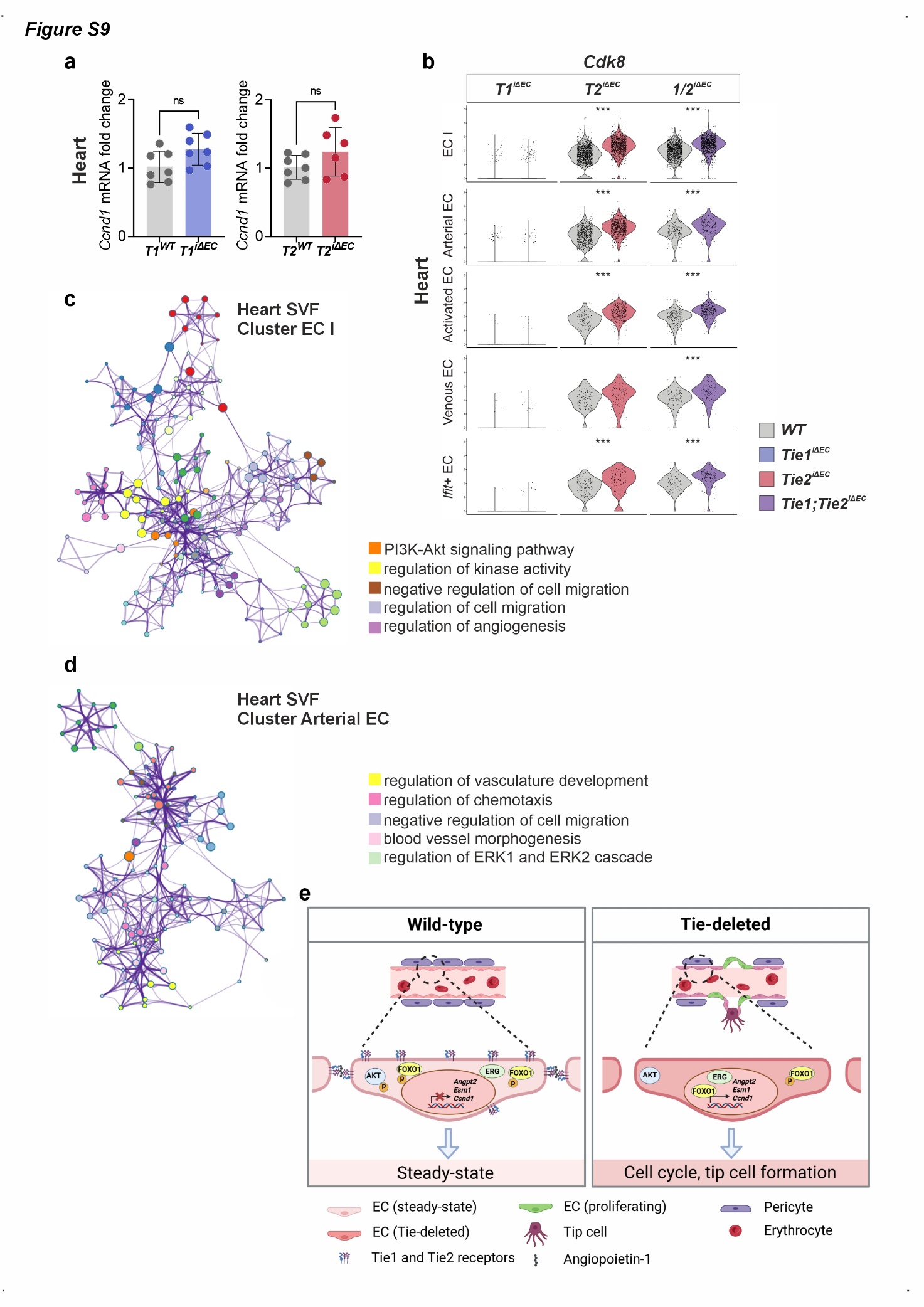
**

**SUPPLEMENTAL FIGURE LEGENDS**

**Figure S1. Deletion of *Tie1* or *Tie2* increases proliferation and sprouting of cardiac endothelial cells.** (a) Schematic of the genetic constructs used in transgenic mouse strains. (b) and (c) qRT-PCR analysis of *Tie1* and *Tie2* expression in kidney and lung total lysates, respectively, from *Tie1^iΔEC^* and *Tie2^iΔEC^* mice, compared to the respective littermate controls, *Tie1^WT^* and *Tie2^WT^*, respectively. (n=5-8 per group). (d) qRT-PCR analysis of *Tie1* and *Tie2* expression in heart, kidney, and lung total lysates, from *Tie2^iΔEC^* and *Tie1^iΔEC^* mice, respectively, compared to the respective littermate controls. (n=3 per group). (e) Quantification of EdU/ERG nuclei in the heart, kidney and lung of *Tie1^iΔEC^* and *Tie2^iΔEC^* mice (compared to *Tie1^WT^* and *Tie2^WT^* controls). Representative images and quantification from *Tie1/Tie2*-double deleted heart kidney and lung see in **Fig. 1c, d**. (n=4-7 per group). (f) and (g) Representative images of sections from *Tie1/Tie2*-double deleted kidney and lung, immunofluorescently stained for CD31 and αSMA. Arrows indicate examples of the branchpoints, as described in the **Materials and Methods** section. Note, representative images of CD31/αSMA-stained sections of *Tie1/Tie2*-double deleted heart are shown in **Fig. 1e**. Representative images of *Tie1*- or *Tie2*-deleted heart, kidney or lung are not shown as the staining patterns are similar to the ones from *Tie1/Tie2*-deleted mice. Note, quantifications of branchpoints from **Fig. 1e, S1f, g** are shown in **Fig. 1f**. (h) Quantification of vascular area from the images in **Fig. 1e, S1f, g**, including the image data from *Tie1*- or *Tie2*-deleted heart, kidney, and lung, which are not shown. For statistics in all the shown bar graphs 2-tailed Student’s *t*-test was used. (n=3-11 per group). Each dot represents average value per mouse. **P<0.05; ***P<0.001*.

**Figure S2. *Tie2* deletion increases cardiac vessel branchpoints in SHAM and TAC conditions.** (a) Representative images of CD31-stained cardiac sections after *Tie2* deletion under SHAM or TAC conditions. Arrows indicate examples of branchpoints. (b) Quantifications of branchpoints and vascular area from the images as in (a). (n=3-6 per group). (c) qRT-PCR analysis of *Angpt2* and *Esm1* mRNAs in whole hearts obtained from TAC model. (n=3-6 per group). (d) Heart weight-to-body weight ratio after *Tie2* deletion for one month in SHAM or TAC conditions. (n=3-6 per group). (e) and (f) Representative images and quantification of Sirius Red-stained cardiac sections obtained from the TAC model. This data indicates a lack of fibrotic changes in *Tie2*-deleted hearts under SHAM/TAC conditions. (n=3-5 per group). For statistics on graphs (b) – (d) and (f) one-way ANOVA followed by Dunnett’s *post hoc* test was used. Each dot represents average value per mouse. **P<0.05; **P<0.01; ***P<0.001*.

**Figure S3. *Tie1* and/or *Tie2* deletions do not cause cardiac hypertrophy, fibrosis, or inflammatory cell infiltration.** (a) Heart weight-to-body weight ratio after *Tie1*, *Tie2* or *Tie1/Tie2* deletions for one month. (n=6-7 per group). (b) and (c) Quantification and representative images of Sirius Red-stained cardiac sections indicating the lack of fibrotic changes in our genetic models. (n=3-5 per group). (d) – (f) Representative images and quantification of CD45**^+^** cells in the heart, kidney, or lung after *Tie1/Tie2* deletion. (n=3 per group). For statistics in (a), (b) and (f) 2-tailed Student’s *t*-test was used. Each dot represents average value per mouse. ***P<0.01.*

**Figure S4. Stable silencing of *TIE1* and *TIE2* in cultured HUVECs results in activation of different biological pathways.** (a) Western blotting of total HUVEC lysates after stable silencing of *TIE1* or *TIE2* mRNAs using lentivirus-encoded shRNAs. (b) and (c) GO enrichment analysis of DEGs activated as a result of stable silencing of *TIE2* or *TIE1* in HUVECs. Clustering was performed using Metascape (https://metascape.org/gp/index.html#/main/step1) and visualized using Cytoscape (v. 3.10.2). (d) Quantification of length-to-width ratio for the **Supplemental Videos S1-S3**. (n=6-10 per group). For statistics on graphs one-way ANOVA followed by Dunnett’s *post hoc* test was used. Each dot represents average value per mouse. ****P<0.001.*

**Figure S5. EC cluster-determining genes and UMAP plots from the *Tie1^iΔEC^*, *Tie2^iΔEC^, Tie1/Tie2^iΔEC^* and control hearts.** (a) Violin plots showing expression distribution of cluster-determining genes for five major EC cardiac clusters in three genotypically-defined groups of mice. For cluster determination, we used control (*Cdh5-BAC-CreER-negative*) mice from each of the strains, i.e., *Tie1^WT^*, *Tie2^WT,^* and *Tie1;Tie2^WT^*. (b) UMAP plots of CD31-enriched fraction of cells from the hearts of *Tie1^iΔEC^* mice and their littermate controls *Tie1^WT^*. Analysis parameters and cluster markers are shown in **Supplemental Tables 4** and **6**, respectively. More data regarding differentially expressed genes and associated pathway analysis for this tissue and genetic model can be found in **Supplemental Tables 11, 17** and **18**. (c) UMAP plots of SVF fraction of cells from the hearts of *Tie2^iΔEC^* mice and their littermate controls *Tie2^WT^*. Analysis parameters and cluster markers are shown in **Supplemental Tables 4** and **7**, respectively. More data regarding differentially expressed genes and associated pathway analysis for this tissue and genetic model can be found in **Supplemental Tables 12, 19** and **20**. (d) and (e) qRT-PCR analysis of *Angpt2* and *Esm1* mRNA expression in total lysates from the hearts of *Tie1^iΔEC^* and *Tie2^iΔEC^* mice, compared to the respective littermate controls *Tie1^WT^* and *Tie2^WT^*. (n=6-7 per group). For statistics in (d) and (e) 2-tailed Student’s *t*-test was used. Each dot represents average value per mouse. ***P<0.01; ***P<0.001.*

**Figure S6. Expression of tip cell markers and *Ccnd1* in kidney upon *Tie*-deletion.** (a) Schematic summary of the tissue processing for scRNAseq following the experimental ending. (b) UMAP plots showing cluster distribution in CD31-enriched cells from the kidney. Analysis parameters and cluster markers are shown in **Supplemental Tables 4** and **8**, respectively. More data regarding differentially expressed genes and associated pathway analysis for this tissue and genetic model can be found in **Supplemental Tables 13, 21** and **22**. (c) Violin plots showing expression of the four tip cell markers in major renal EC clusters upon *Tie2* deletion. Violin plots have the same scale of [0, 6]. Significance is indicated based on results from differential gene expression analysis, shown in detail in **Supplemental Table 13**. (d) and (e) qRT-PCR analysis of *Angpt2* and *Esm1* mRNA expression in total lysates from the kidney of three genotypes, compared to the respective littermate controls. (n=6-7 per group). (f) Violin plots showing expression of the cell cycle regulator *Ccnd1* and proliferation-related gene *Cdk8* in major renal EC clusters upon *Tie2* deletion. For statistics in (d) and (e) 2-tailed Student’s *t*-test was used. For scRNAseq data, Wilcoxon Rank Sum test was used. Each dot represents average value per mouse. **P<0.05; **P<0.01; ***P<0.001.*

**Figure S7. Expression of tip cell markers and *Ccnd1* in lung upon *Tie*-deletion.** (a) Schematic of the tissue processing for scRNAseq following the experimental ending. (b) UMAP plots showing cluster distribution in CD31-enriched cells from lung. Cluster names are shown below. Analysis parameters and cluster markers for this dataset can be found in **Supplemental Tables 4** and **9**, respectively. More data regarding differentially expressed genes and associated pathway analysis for this tissue and genetic model can be found in **Supplemental Tables 14, 23** and **24**. (c) Violin plots showing expression of the four tip cell markers in major pulmonary EC clusters upon *Tie2* deletion. Violin plots are created using the same scale of [0, 6]. Significance is determined based on differential gene expression analysis, for which results are shown in detail in **Supplemental Table 14**. (d) and (e) qRT-PCR analysis of *Angpt2* and *Esm1* mRNA expression in total lysates from the lung of three genotypes, compared to the respective littermate controls. (n=6-7 per group). (f) Violin plots showing expression of the cell cycle regulator *Ccnd1* and proliferation-related gene *Cdk8* in major pulmonary EC clusters upon *Tie2* deletion. For statistics in (d) and (e) 2-tailed Student’s *t*-test was used. For scRNAseq data, Wilcoxon Rank Sum test was used. Each dot represents average value per mouse. **P<0.05.*

**Figure S8. Nuclear localization of key endothelial transcription factors FOXO1 and ERG indicates tissue-specific effect of *Tie* deletions.** (a) and (b) Lung representative images and quantification of FOXO1 overlapped with ERG. For description of the quantification see legend to **Fig. 2j** and **Materials and methods**. (n=4 per group). (c) Distribution of cardiac ECs based on nuclear ERG accumulation. Shown are the representative images of cardiac sections stained with anti-ERG antibody. Counterstaining was done with DAPI. (d) Quantification from (c). Detailed description of quantification is provided in **Materials and methods** sections. (n=4-7 per group). (e) and (f) Distribution of renal and pulmonary ECs based on nuclear ERG accumulation. Representative images are not shown. Quantification was done in the same way as for the cardiac ECs (see (d) and **Materials and methods** for the details). (n=6-7 per group). For statistics in (b), (d), (e) and (f) 2-tailed Student’s *t*-test was used. Each dot represents average value per mouse. **P<0.05; **P<0.01; ***P<0.001.*

**Figure S9. Tie deletions lead to activation of angiogenesis-associated steps.** (a) RT-qPCR analysis of *Ccnd1* mRNA expression in the total heart lysates of *Tie1^iΔEC^* and *Tie2^iΔEC^* mice, compared to the respective littermate controls *Tie1^WT^* and *Tie2^WT^*. (n=6-7 per group). (b) Violin plots showing expression of the proliferation-related gene *Cdk8* in five major cardiac EC clusters across three genotypes. Violin plots are created using the same scale of [0, 5]. Significance is indicated based on differential gene expression analysis, for which results can be found in **Supplemental Tables 10-12.** (c) and (d) GO enrichment analyses for DEGs upregulated in *Tie1;Tie2^iΔEC^* cardiac ECs, compared to *Tie1;Tie2^WT^* cardiac ECs (data are obtained in scRNAseq analysis). Shown are the enrichments for the two major EC clusters EC I and Arterial EC. For the analysis we used DEGs with FDR<0.05. The analysis was performed using Metascape and visualized in Cytoscape, as explained in the legend to **Fig. S4b, c**. (e) Schematic showing chain of events in cardiac ECs upon Tie deletion. In wild-type mice (left panel) ECs express TIE1 and TIE2 receptors that maintain steady-state via, in part, activation of PI3K-AKT axis. AKT phosphorylates FOXO1, thereby promoting its accumulation and degradation in the cytoplasm. ERG preferentially stays in cytoplasm. In contrast, TIE2 or TIE1/TIE2 deletion (right panel) leaves PI3K-AKT axis inactive. AKT does not phosphorylate FOXO1, which is then accumulated in the nuclei, where it transactivates transcription of a number of genes including *Angpt2*, *Esm1* and *Ccnd1*. Similar events are known for the ERG transcription factor, although phosphorylation may not be involved in its nuclear translocation in ECs. Elevated expression of tip cell-marker and proliferation-related genes increases frequency of branch point formation and the number of proliferating ECs. For statistics in (a) 2-tailed Student’s *t*-test was used. Each dot represents average value per mouse. ****P<0.001*.

**Video S1. HUVECs treated with lentivirus-shScramble**

**Video S2. HUVECs treated with lentivirus-shTie1 (clone A)**

**Video S3. HUVECs treated with lentivirus-shTie2 (clone A)**

**Supplemental Table 1. Echocardiography data of cardiac parameters after *Tie1, Tie2 or Tie1/Tie2* gene deletions in homeostatic or ischemic (TAC vs. SHAM) conditions.** Data obtained from *Tie1*, *Tie2* or *Tie1/Tie2* deletion models in homeostatic conditions are shown in the upper half of the table. The data obtained after *Tie2* deletion in SHAM or TAC are shown in the lower half of the table. For statistics, 2-tailed Student’s *t*-test was used.

**Supplemental Table 2.** Cell type frequencies in all datasets.

**Supplemental Table 3.** EC percentages in the clusters of proliferating cells.

**Supplemental Table 4.** Parameters used for quality control, filtering, normalization, integration, and clustering of the different single-cell RNA sequencing datasets (heart, kidney or lung) shown in this manuscript.

**Supplemental Table 5.** Cluster markers calculated for the murine *Tie1/Tie2* EC double-deletion model heart stromal vascular fraction single-cell RNA sequencing dataset.

**Supplemental Table 6.** Cluster markers calculated for the murine *Tie1* EC deletion model heart EC single-cell RNA sequencing dataset.

**Supplemental Table 7.** Cluster markers calculated for the murine *Tie2* EC deletion model heart stromal vascular fraction single-cell RNA sequencing dataset.

**Supplemental Table 8.** Cluster markers calculated for the murine *Tie2* EC deletion model kidney EC single-cell RNA sequencing dataset.

**Supplemental Table 9.** Cluster markers calculated for the murine *Tie2* EC deletion model lung EC single-cell RNA sequencing dataset.

**Supplemental Table 10.** Differentially expressed genes in heart stromal-vascular fraction cell types in mice with *Tie1/Tie2* EC double-deletion versus wild-type mice.

**Supplemental Table 11.** Differentially expressed genes in heart endothelial cell types and pericytes in mice with *Tie1* EC deletion versus wild-type mice.

**Supplemental Table 12.** Differentially expressed genes in heart stromal-vascular fraction cell types in mice with *Tie2* EC deletion versus wild-type mice.

**Supplemental Table 13.** Differentially expressed genes in kidney ECs in mice with *Tie2* EC deletion versus wild-type mice.

**Supplemental Table 14.** Differentially expressed genes in lung ECs in mice with *Tie2* EC deletion versus wild-type mice.

**Supplemental Table 15.** Enrichment of downregulated GO terms in heart stromal-vascular fraction cell types in mice with *Tie1/Tie2* EC double-deletion versus wild-type mice.

**Supplemental Table 16.** Enrichment of upregulated GO terms in heart stromal-vascular fraction cell types in mice with *Tie1/Tie2* EC double-deletion versus wild-type mice.

**Supplemental Table 17.** Enrichment of downregulated GO terms in heart endothelial cell types and pericytes in mice with *Tie1* EC deletion versus wild-type mice.

**Supplemental Table 18.** Enrichment of upregulated GO terms in heart endothelial cell types and pericytes in mice with *Tie1* EC deletion versus wild-type mice.

**Supplemental Table 19.** Enrichment of downregulated GO terms in heart stromal-vascular fraction cell types in mice with *Tie2* EC deletion versus wild-type mice.

**Supplemental Table 20.** Enrichment of upregulated GO terms in heart stromal-vascular fraction cell types in mice with *Tie2* EC deletion versus wild-type mice.

**Supplemental Table 21.** Enrichment of downregulated GO terms in kidney ECs in mice with *Tie2* EC deletion versus wild-type mice.

**Supplemental Table 22.** Enrichment of upregulated GO terms in kidney ECs in mice with *Tie2* EC deletion versus wild-type mice.

**Supplemental Table 23.** Enrichment of downregulated GO terms in lung ECs in mice with *Tie2* EC deletion versus wild-type mice.

**Supplemental Table 24.** Enrichment of upregulated GO terms in lung ECs in mice with *Tie2* EC deletion versus wild-type mice.

**Supplemental Table 25.** Antibodies used in the study.

**Supplemental Table 26.** Primers and Taqman probes.

**Supplemental Table 27.** TRC clones used in silencing,

**SUPPLEMENTAL MATERIALS AND METHODS**

**Mice and gene deletions**

All animal experiments were approved by the Committee for Animal Experiments of the District of Southern Finland.

The *Tie1^fl/fl^* [1], *Tie2^fl/fl^* [2], *Tie1^fl/fl^*;*Tie2^fl/fl^* [3], and *Cdh5-BAC-CreER^T2^* [4], mouse strains (described previously) were used on C57BL/6J genetic background. Cre-mediated excision deletes the first exon of *Tie2* or *Tie1* and 3524 nt or 1428 nt, respectively, of the upstream nucleotide sequence preceding the transcription start site. To induce Cre-mediated gene deletions, each mouse was administered tamoxifen (Sigma-Aldrich; 20 mg/mL in 100 µL corn oil) via oral gavage for five consecutive days. Mice were provided with water and food *ad libitum* and maintained on a 12 h/12 h light/dark cycle at 22 ± 2 °C with a relative humidity of 55 ± 10%. Only male mice were used in the studies. Age matched Cre-negative mice were used as controls. Poor health was a predefined exclusion criterion; no mice met this criterion. Only male mice were used in this study.

Here, we want to briefly explain how we denote gene-deleted mice. In line with common practice in the field, we have used simplified notation, in which only the allele that is floxed or deleted is indicated. Thus, when we refer to *Tie2^iΔEC^*, it is means that *Tie1* is wild-type, and when we refer to *Tie1^iΔEC^*, *Tie2* is wildtype. Therefore, if they are both mentioned, that means they are both floxed; since when both alleles were floxed, they are deleted simultaneously.

**Cell lines and primary cells**

293T cells were purchased from ATCC. HUVECs were purchased from PromoCell. 293T cells were cultured in DMEM, supplemented with 10% FCS and L-glutamine. HUVECs were cultured in Basal Medium MV (PromoCell, cat # C-22220), supplemented with Supplement Pack GM MV (cat # C-39220) and were used for experiments in passage 4 to 6. When indicated, puromycin (2 µg/mL) was added to the culture medium, as a selection antibiotic.

**EdU treatment**

EdU (Invitrogen), dissolved in PBS (to the concentration of 5 mg/mL) and injected i.p. 0.1 mL per mouse every 12 hours during the final three days of the housing period (4 weeks). For the detection of EdU incorporated into the host DNA we used Click-IT^TM^ EdU Alexa FluorTM 594 Imaging Kit (Invitrogen; cat. # C10339).

**Transverse Aortic Constriction (TAC) model**

Mice were anesthetized with 2% isoflurane mixed with 0.5 -1.0 L/min 100% O_2_. During the surgical procedure, anesthesia was maintained at 1.5-2% isoflurane with 0.5 - 1.0 L/min 100% O_2_. Ligation of the transverse aorta was performed using a 7.0 silk suture with the aid of 27G blunt needle. For post-operative recovery, buprenorphine (0.1 mg/kg) was used as a pain medication, injected intraperitoneally. The achieved constriction of the aorta was approximately 30%. To note, our analysis in this model was focused solely on the heart (without analysis of kidney or lung) as this was the organ undergoing experimental treatment.

**Echocardiography**

To analyze cardiac function and ventricle dimensions, two-dimensional echocardiography images were acquired (Vevo 2100 Ultrasound, FUJIFILM Visual Sonics). The LV internal diameter, LV posterior wall thickness, and interventricular septum thickness at end-systole and end-diastole were measured in M-mode along the parasternal short axis view and analyzed by Simpson’s modified method [5].

**Immunofluorescence staining of heart, kidney, and lung sections**

Frozen 10 µm sections were air-dried (30 min, RT), fixed with PFA (4% in PBS), blocked with blocking reagent from TSA INDIRECT kit (PerkinElmer) and incubated with primary antibodies overnight. The unbound antibodies were washed away with the wash solution (150 mM NaCl, 100 mM Tris, 0.05% Tween-20, pH 7.5), and the sections were incubated with the secondary antibodies, conjugated with Alexa fluorophores for 1 h at RT. The sections were washed, nuclei were stained with DAPI (2.5 µg/mL), and the sections were mounted with Vectashield for fluorescence (Vector Laboratories). Microscopic images were analyzed using ImageJ software (NIH) and/or AngioTool64 (NIH, version 0.6a (02.18.14)).

**Microscopy of immunofluorescence stainings**

The samples were analyzed with a Zeiss LSM 780 confocal microscope equipped with digital camera (Carl Zeiss AG, Oberkochen, Germany; objectives Plan-Apochromat 10x/0.45 M27, Plan-Apochromat 20x/0.8 M27 and Plan-Apochromat 40x/1.4 Oil DIC M27.

**Sirius Red staining of cardiac sections to visualize fibrosis**

The staining was done according to the protocol: [https://ihcworld.com/2024/01/26/sirius-red-staining-protocol-for-collagen/.](https://ihcworld.com/2024/01/26/sirius-red-staining-protocol-for-collagen/) We used cardiac sections frozen in O.C.T. for Sirius Red and Weigert’s haematoxilin, which were purchased from Sigma-Aldrich.

**Production of lentiviruses encoding shRNAs and cell treatment**

Lentiviral production was done essentially as described [6]. In brief, packaging plasmid vectors CMVg and CMV_∆_8.9 and target plasmid (pLKO.1-shTie1, pLKO.1-shTek or pLKO.1-Scrambled, selected from TRC Library Database (https://portals.broadinstitute.org/gpp/public/) were co-transfected into 293T cells. Lentivirus-containing supernatants were harvested after 48 h and again after subsequent 24 h, combined and concentrated by ultracentrifugation. For the cell treatment, subconfluent endothelial cell cultures (HUVEC, 80-90%) were incubated with lentiviral preps in endothelial cell medium (overnight). Lentiviral medium was then replaced with fresh endothelial medium and incubated further for various periods of time (1 to 4 days). Unless otherwise indicated, puromycin (selection agent) was added to a final concentration of 2 µg/mL. List of TRC clones used can be found in **Supplemental Table 27**.

**Boyden chamber transwell assay**

HUVECs were treated with shTie2, shTie1 or Scr lentiviruses, selected with puromycin, detached, and 30000 cells were seeded onto each 8-μm pore size polycarbonate membranes (Costar, 3422), coated with a mixture of fibronectin (Sigma-Aldrich, F0895, 10 μg/mL) and laminin-211 and 511 isoforms (1 μg/mL each; BioLamina). Cells were allowed to migrate for 3 h at +37^o^C cell culture incubator. Non-migrated cells were removed with cotton swabs and migrated cells were fixed and stained with Hoechst 33258. Fluorescent images were quantified using the ImageJ program (NCBI).

**Video recording of cell motility**

HUVECs were treated with lentiviruses as described in Boyden chamber transwell assay section, detached with trypsin and plated onto the uncoated plastic surface of a 48-well plate at subconfluent cell density in full endothelial medium without puromycin for 38 h in Cell-IQ live cell imaging system, equipped with 10x objective lens (CM Technologies, Tampere, Finland). Images were acquired every 15 min. Sets of images were then stacked in ImageJ to create .avi movies. The movies were compressed to .mov files in Adobe Premiere Pro CC 2025.

**RNA profiling by qRT-PCR**

Total RNA was isolated from cultured cells or tissues using NucleoSpin RNA kit (Macherey-Nagel) and the first DNA strand was synthesized using iScript^TM^ cDNA synthesis kit (Bio-Rad). PCR was run using CFX Opus 96 (Bio-Rad). List of primers and TaqMan probes is in **Supplemental Table 26**.

**Isolation of cardiac, renal and pulmonary ECs or total cardiac SVF for scRNAseq**

Tie deleted and the corresponding control mice were sacrificed by terminal anesthesia followed by cervical dislocation. Hearts were minced using scissors and enzymatically dissociated in 1 mg/mL each of lyophilized collagenase type I, II and IV (Gibco) at 37°C for 25 min. The suspensions containing cell clumps were repeatedly pipetted to achieve single-cell suspension. RBC lysis buffer (Sigma-Aldrich) was used to lyze the leftover erythrocytes. Cell suspension was further filtered through the series of nylon mesh of 70, 40, 35 and 20 µm pore sizes. Single-cell suspensions were washed using DPBS with 2% heat-inactivated FCS and resuspended in DPBS containing 0.04% BSA. This sample represented stromal-vascular fraction. To obtain cardiac cell suspension enriched for ECs, the cells were incubated with mouse Fc blocker (rat-anti-mouse CD16/CD32 (BD Biosciences) for 5 min followed by the antigen-specific antibody fluorescent conjugates for 30 min (list of the antibodies is in **Supplemental Table 25**). The live cardiac ECs (CD31^+^ CD45^-^ Ter119^-^ CD140a^-^ DAPI^-^) were sorted in FACS Aria II (BD Biosciences). After the FACS step, cardiac ECs were washed using DPBS with 2% heat-inactivated FCS and were resuspended in DPBS containing 0.04% of BSA.

The cell suspensions from kidney and lung were prepared as described earlier [7] obtained by enzymatic digestion, enriched for ECs by FACS-sorting and prepared for scRNAseq analysis in a similar fashion.

**Single-cell RNA sequencing**

Isolated cardiac, lung or kidney cells from 2–3 individual *Tie2^WT^*, *Tie2^iΔEC^*, *Tie1^WT^*, *Tie2^WT^*, and *Tie1;Tie2^iΔEC^* mice were pooled together, resuspended in DPBS containing 0.04% BSA as one sample per genotype, and then processed using a Chromium Single Cell 3′ RNA sequencing system (Reagent Kit v3.1, 10x Genomics). Cells were loaded into a Chromium Single Cell Chip aiming at capturing 10,000 cells per sample. Sample libraries were sequenced using an Illumina NovaSeq 6000 system S1 flow cell with the following read lengths: read 1, 28 bp; i7 index, 8 bp; i5 index, 0 bp; and read 2, 89 bp.

Raw sequencing data were processed using the Cell Ranger pipeline (10x Genomics). To reduce any potential bias due to ambient RNA contamination, we performed ambient RNA removal using CellBender V0.3.0 with default parameters. Next, the data were imported to an R environment V4.5.0 using the Seurat V5.1.0 package, filtering out cells with less than 200 features and features detected in less than 3 cells. The cells were further filtered in a sample-dependent manner for the number of features, mitochondrial RNA-%, and ribosomal RNA-%. The exact filtering parameters for each sample are shown in **Supplemental Table 4**. The Seurat package was further utilized for log normalization of the samples, identifying the 2000 most variable features, determining integration anchors between samples, and ultimately integrating the samples in a tissue- and deletion-dependent manner. The normalization and sample integration details can be found from **Supplemental Table 4**. Next, the data were scaled regressing for RNA counts, mitochondrial RNA-%, and ribosomal RNA-%, and principal component analysis was performed based on the 2000 most variable features for each integrated dataset. Clusters were calculated for each integrated dataset using varying dimensions and resolution (**Supplemental Table 4**). The clusters were visualized using Uniform Manifold Approximation and Projection (UMAP). The cluster number for each dataset is shown in **Supplemental Table 2**. Cluster markers were calculated for each cluster in each dataset using the FindMarkers()-function (min.pct= 0.25) in the Seurat package. For finding cluster markers, only the control samples for each dataset were used to avoid treatment-induced bias. The clusters were labelled based on literature including similar data and cell types, as well as our own classification. Clusters labelled as “damaged” included a lower expression of most genes in comparison to all other clusters. These cells were removed from further analyses. The labelled clusters with their corresponding cluster markers can be found from **Supplemental Tables 5-9**.

The individual samples of each integrated dataset were downsampled to correspond to the sample with the lowest cell number (per dataset). Then, the samples were merged back into one Seurat object using the merge()-function. Next, differential gene expression analysis was performed using the FindMarkers()-function (logFC threshold= 0.25) using default settings. The differentially expressed genes for each comparison can be found from **Supplemental Tables 10-14**. Genes of interest were visualized using the VlnPlot()-function, with the scale set to a range of [0, 5] (cardiac datasets) or [0, 6] (renal and lung datasets) for all graphs, allowing easier comparison of expression levels.

**Microscopy of immunofluorescence stainings**

The samples were analyzed with a Zeiss LSM 780 confocal microscope equipped with digital camera (Carl Zeiss AG, Oberkochen, Germany; objectives Plan-Apochromat 10x/0.45 M27, Plan-Apochromat 20x/0.8 M27 and Plan-Apochromat 40x/1.4 Oil DIC M27.

**Quantification of nuclear FOXO1 in Fig. 2i, j** and **S8a, b**

For quantification, we used Fiji software (ImageJ, v. 1.54p, NCBI). First, we selected and quantified all nuclear ERG from fluorescence intensity (F.I.) per µm^2^ for each individual nucleus. These values were divided by a background signal from ERG staining representing a total ERG F.I. (from the whole image) divided by the image area (µm^2^). These data indicate fold change of the nuclear ERG over the background ERG. We used 1.5-fold change as a threshold to remove low ERG-expressing nuclei that can include significant portion of non-ECs. In parallel, using the same approach, we quantified nuclear FOXO1 as a fold-change over the background FOXO1. For the final calculations, we used only nuclei that showed ERG >1.5 fold over the ERG background. These nuclei in the sample represent 100%. We then counted the percentage of those nuclei that had FOXO1 expression more than 2 (or more) times the background FOXO1 staining.

**Quantification of nuclear ERG in Fig. S8c-f**

For quantification we selected all DAPI-stained nuclei and determined ERG fluorescence intensity (F.I.) and nuclear area (µm^2^) and calculated F.I. per area unit (µm^2^) for each nucleus. In parallel, we quantified “total” ERG that was divided by the total image area (in µm^2^). This value was used as a “background ERG”. The nuclear ERG F.I. per unit of area values (as explained above) were then divided by the “background ERG” value, producing the fold increase of the nuclear ERG for each individual nucleus. The higher fold increase, the higher accumulation of ERG in the nucleus. Data are presented as bar graphs showing percentage of the nucleus with ERG exceeding “background ERG” 2x, 3x, 4x or 5x, compared to the total ERG+ nuclei (all nuclei where ERG is higher than the background).

**Definition and quantification of branchpoints**

We used Pecam1 (CD31)-stained sections. Images were acquired using confocal microscopy with 20x magnification. Branchpoints in heart and kidney samples were quantified manually and the lung samples were quantified using AngioTool. We defined a branch point as a site where three or more vessels converge. Typically, two of these vessels represent pre-existing capillaries, while the third is a newly sprouted vessel. In manual quantification we carefully analyzed the image and marked all branching spots on capillaries, i.e. areas where one can find a side branch sprouting from the trunk. Every such area was marked with a dot, and the number of dots per image was counted. Manual quantification (in a blinded manner) allowed for a more precise determination of the branchpoints, as AngioTool sometimes yields false positive branch point counts due to background. It should be noted that the manual quantification was possible in the heart and kidney due to the relatively low density of the vascular network in these organs. In contrast, vascular density in the lung (and the number of branchpoints) is about 10-fold higher than in the heart and kidney, thus making it unrealistic to count branchpoints manually. Therefore, we used AngioTool with its default parameters to count branch points in the lung.

**Statistics**

Statistical significance was evaluated either using an unpaired 2-tailed Student’s *t-*test or a one-way ANOVA, followed by a Dunnett (two-sided) post-hoc test, with p<0.05 regarded as significant. A Welch correction was applied in case of inequal variances. The scRNAseq data was compared using the default Wilcoxon Rank Sum test (default) in FindMarkers function of Seurat. The results are presented as mean values ± SD, unless otherwise indicated.

**Bioinformatics**

Enrichment Analysis was done using Metascape [8]. To visualize GO terms network, we used Cytoscape (version: 3.10.2) software [9].

Raw and filtered scRNAseq sequencing files are available on GEO (accession number GSE297297, with reviewer token snshueamxlenruz).

The list of antibodies, primers and shRNAs used in the study are shown in **Supplementary Tables 25-27**.

**REFERENCES**

1. Korhonen EA, Lampinen A, Giri H, et al (2016) Tie1 controls angiopoietin function in vascular remodeling and inflammation. J Clin Invest 126:3495–510. https://doi.org/10.1172/JCI84923

2. Savant S, La Porta S, Budnik A, et al (2015) The Orphan Receptor Tie1 Controls Angiogenesis and Vascular Remodeling by Differentially Regulating Tie2 in Tip and Stalk Cells. Cell Rep 12:1761–73. https://doi.org/10.1016/j.celrep.2015.08.024

3. Anisimov A, Fang S, Hemanthakumar KA, et al (2023) The angiopoietin receptor Tie2 is atheroprotective in arterial endothelium. Nature cardiovascular research 2:307–321. https://doi.org/10.1038/s44161-023-00224-y

4. Okabe K, Kobayashi S, Yamada T, et al (2014) Neurons limit angiogenesis by titrating VEGF in retina. Cell 159:584–96. https://doi.org/10.1016/j.cell.2014.09.025

5. Kivelä R, Hemanthakumar KA, Vaparanta K, et al (2019) Endothelial Cells Regulate Physiological Cardiomyocyte Growth via VEGFR2-Mediated Paracrine Signaling. Circulation 139:2570–2584. https://doi.org/10.1161/CIRCULATIONAHA.118.036099

6. Lois C, Hong EJ, Pease S, et al (2002) Germline transmission and tissue-specific expression of transgenes delivered by lentiviral vectors. Science 295:868–72. https://doi.org/10.1126/science.1067081

7. Karaman S, Paavonsalo S, Heinolainen K, et al (2022) Interplay of vascular endothelial growth factor receptors in organ-specific vessel maintenance. Journal of Experimental Medicine 219:. https://doi.org/10.1084/jem.20210565

8. Zhou Y, Zhou B, Pache L, et al (2019) Metascape provides a biologist-oriented resource for the analysis of systems-level datasets. Nat Commun 10:1523. https://doi.org/10.1038/s41467-019-09234-6

9. Shannon P, Markiel A, Ozier O, et al (2003) Cytoscape: a software environment for integrated models of biomolecular interaction networks. Genome Res 13:2498–504. https://doi.org/10.1101/gr.1239303
